# Characterization of a synthetic human LINE-1 retrotransposon *ORFeus*-Hs

**DOI:** 10.1186/1759-8753-2-2

**Published:** 2011-02-14

**Authors:** Wenfeng An, Lixin Dai, Anna Maria Niewiadomska, Alper Yetil, Kathryn A O'Donnell, Jeffrey S Han, Jef D Boeke

**Affiliations:** 1 Department of Molecular Biology and Genetics, Johns Hopkins University School of Medicine, Baltimore, MD 21205, USA; 2 School of Molecular Biosciences, Washington State University, Pullman WA 99164, USA; 3 Stanford University School of Medicine, Stanford, CA 94305, USA; 4 Carnegie Institution of Washington, 3520 San Martin Drive, Baltimore, MD 21218, USA

## Abstract

Long interspersed elements, type 1(LINE-1, L1) are the most abundant and only active autonomous retrotransposons in the human genome. Native L1 elements are inefficiently expressed because of a transcription elongation defect thought to be caused by high adenosine content in L1 sequences. Previously, we constructed a highly active synthetic mouse L1 element (*ORFeus*-Mm), partially by reducing the nucleotide composition bias. As a result, the transcript abundance of *ORFeus*-Mm was greatly increased, and its retrotransposition frequency was > 200-fold higher than its native counterpart. In this paper, we report a synthetic human L1 element (*ORFeus*-Hs) synthesized using a similar strategy. The adenosine content of the L1 open reading frames (ORFs) was reduced from 40% to 27% by changing 25% of the bases in the ORFs, without altering the amino acid sequence. By studying a series of native/synthetic chimeric elements, we observed increased levels of full-length L1 RNA and ORF1 protein and retrotransposition frequency, mostly proportional to increased fraction of synthetic sequence. Overall, the fully synthetic *ORFeus*-Hs has > 40-fold more RNA but is at most only ~threefold more active than its native counterpart (L1_RP_); however, its absolute retrotransposition activity is similar to *ORFeus*-Mm. Owing to the elevated expression of the L1 RNA/protein and its high retrotransposition ability, *ORFeus*-Hs and its chimeric derivatives will be useful tools for mechanistic L1 studies and mammalian genome manipulation.

## Background

The human genome is littered with transposable element sequences; some are mere fossil records of ancient insertion events, whereas others remain active. Of these active elements, the long interspersed elements, type 1 (LINE-1 or L1) remain among the most active, and are capable of autonomous retrotransposition [1] and of providing enzymatic activities for the non-autonomous retrotransposition of short interspersed nucleotide elements (SINE) such as *Alu *elements [2]. Full-length versions of L1 elements are approximately 6 kb long, and consist of a 5' (untranslated region) UTR containing an internal promoter sequence, two open reading frames (ORFs), ORF1 and ORF2, and a 3'UTR followed by a poly(A) tail encoded in the DNA [3-8]. The L1 ORF1 protein (ORF1p) is a non-specific nucleic acid binding protein with nucleic acid chaperone activity [9-12]. The ORF2 protein (ORF2p) is responsible for the catalytic activity necessary for retrotransposition, and contains both endonuclease and reverse transcriptase activities [13,14].

L1s make up approximately 17% of the human genome. However, despite their abundance, the replication and control mechanisms of these elements are poorly understood, partly because of their low expression levels of messenger (m)RNA and protein [15]. We have previously linked inefficient L1 expression to a transcription elongation defect potentially caused by high adenosine content in the ORFs. We subsequently constructed a synthetic L1, termed *ORFeus*, in which the codons of both ORFs were synonymously optimized, based on a mouse L1 protein sequence [16,17]. This element was at least 200-fold more active for retrotransposition than the native mouse element L1_spa _[18].

In this paper, we describe our use of similar techniques to construct a synthetic human L1 (*ORFeus*-Hs) element and several synthetic/native chimeric L1 elements. Although we observed increased levels of L1 mRNA and ORF1p, the levels of L1 retrotransposition, as measured by two different retrotransposition reporter assays [1,19], were only increased by a maximum of about threefold in this element. We discuss various models to explain the possible restrictions on *ORFeus-*Hs activity. Certain chimeric synthetic/native constructs were higher in activity than the fully synthetic constructs, suggesting that recoding may have abolished a *cis *element(s) or introduced one or more deleterious sequences into *ORFeus-*Hs. Moreover, one of these chimeras produced slightly more mRNA and ORF1 protein compared with *ORFeus-*Hs. *ORFeus*-Hs represents a valuable tool for studying mechanisms of L1 replication and control, particularly at the protein level, by providing nucleic acid and protein markers that can be detected more easily.

## Results

### Construction of ORFeus-Hs and the synthetic/native L1 chimeras

The *ORFeus*-Hs open reading frames were designed using the same principles used to construct murine *ORFeus*, which we now refer to as *ORFeus*-Mm to distinguish it from the main topic of this paper; *ORFeus*-Mm was referred to as smL1 in the original publication [16]. The reading frames were recoded to the preferred codon for each amino acid (that is, 20 codons were used), except where internal restriction sites were strategically positioned to facilitate assembly of the complete synthetic ORFs (see Additional file 1, Figure S1). The synthetic ORFs were fused either to a cytomegalovirus (CMV) promoter-enhancer with a Kozak signal, the native L1 5' UTR promoter, or a combination of both (see Additional file 1, Figure S2 for sequences of these segments). These constructs were also tagged with either enhanced green fluorescent protein (*EGFP-AI*) [19] or neomycin (*Neo-AI*) [1] retrotransposition markers to monitor retrotransposition frequency.

**Figure 1:**
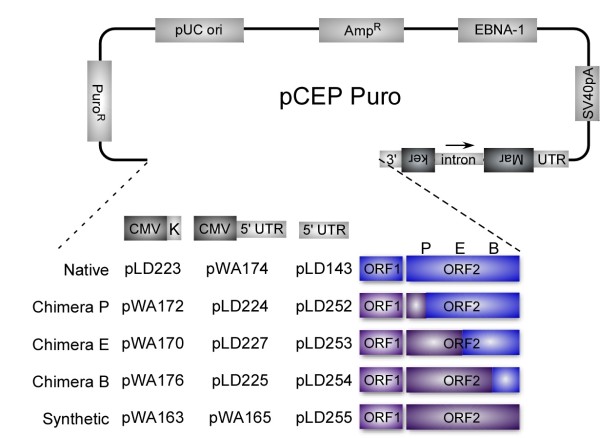
**Schematic representation of native, synthetic and chimeric human L1 elements**. Three sets of such constructs differing from each other at the promoter region are illustrated: the first set carries a cytomegalovirus (CMV) promoter and a Kozak (K) signal, the second set has a dual CMV-L1 5' untranslated region (UTR) promoter, and the third has a 5' UTR promoter only. All elements are cloned in a pCEP-Puro vector backbone. AMP^R ^= ampicillin resistance gene; B, E, P = restriction sites *EcoR*I, *BamH*I and *Pml*I, respectively, at the junctions in various chimeras; EBNA-1 = Epstein-Barr nuclear antigen 1 gene permitting extrachromosomal replication; Intron = human gamma globin intron; Marker = either enhanced green fluorescent protein or neomycin marker; ORF = open reading frame; Puro^R ^= puromycin resistance gene; SV40pA = Simian virus 40 polyadenylation signal. Blue = native sequence; purple = synthetic sequence.

Finally, because synthetic and native elements showed distinct retrotransposition frequencies, and to further study the sequence requirements for L1 retrotransposition, we made several chimeric L1 elements consisting of various combinations of native and synthetic L1 elements (Figure 1; see Additional file 2, Table S1).

### Active retrotransposition by ORFeus-Hs and synthetic/native L1 chimeras

To explore the effects of our sequence manipulations on the levels of L1 retrotransposition, retrotransposition frequency was measured using several independent assays (Table 1). Briefly, the transfected HEK-293T cells were harvested, and EGFP-positive cells were counted by fluorescence-activated cell sorting (FACS) analysis. Retrotransposition levels of the corresponding native L1 sequences with their various promoters (pLD223, pWA174, pLD143) were used as a reference for the other constructs (Table 1, row 1).

**Table 1:** Retrotransposition frequency of *ORFeus*-Hs constructs.

Construct^a^	CMV+Kozak^b^	CMV+ 5" UTR^c^	5" UTR
Fully native	2.8 ± 0.1 (1) [pLD223]	14 ± 0.3 (1) [pWA174]	3.8 ± 0.3 (1) [pLD143]
Chimera-P	5.8 ± 0.3 (2.07) [pWA172]	11.2 ± 0.2 (0.8) [pLD224]	5.1 ± 0.1 (1.34) [pLD252]
Chimera-E	8.8 ± 0.5 (3.14) [pWA170]	12.1 ± 0.3 (0.86) [pLD227]	5.4 ± 0.3 (1.42) [pLD253]
Chimera-B	10.8 ± 0.4 (3.85) [pWA176]	14.3 ± 0.6 (1.02) [pLD225]	6.9 ± 0.4 (1.82) [pLD254]
*ORFeus*-Hs	8.1 ± 0.3 (2.89) [pWA163]	8.1 ± 0.4 (0.58) [pWA165]	4.5 ± 0.2 (1.18) [pLD255]
*ORFeus*-Mm	ND^d^	9.2 ± 0.8 (0.66) [pWA125]	ND

^a^For each construct, its absolute retrotransposition frequency is represented as percentage of enhanced green fluorescence-positive cells, and data are the mean ± SEM of eight independent experiments. The number in the parentheses represents the fold change of retrotransposition frequency relative to native L1_RP _(shown in the first row), and the relevant plasmid name is given in square brackets.

^b^CMV = cytomegalovirus

^c^UTR = untranslated region.

^d^ND = not determined.

Interestingly, the construct containing both the CMV and native L1 promoters exhibited a significantly higher percentage of EGFP-positive cells (14%) than the constructs containing either promoter on its own (2.8% and 3.8% respectively). As for the partially synthetic chimeric L1 constructs, retrotransposition levels appeared to increase as the length of segments of synthetic L1 sequence increased, regardless of which promoter was driving transcription (Table 1, rows 2 to 4). However, when the fully synthetic L1 constructs (pWA163, pWA165, pLD255) were compared with their respective native constructs (pLD223, pWA174, pLD143), retrotransposition levels were variable (Table 1, row 5). The *ORFeus*-Hs construct driven by a CMV promoter alone was about threefold more active than its native L1 counterpart, whereas the *ORFeus*-Hs construct driven by the 5' UTR promoter alone was only slightly more active (~1.2 times) than its native L1 counterpart. Perhaps most unexpectedly, the *ORFeus*-Hs construct driven by both the CMV and 5' UTR promoters was actually less active (0.58 times) than its native L1 counterpart. Thus, the nature of the promoter used strongly influenced the relative retrotransposition frequency of synthetic versions of retrotransposons.

Additionally, it is of interest that in all of the comparisons, the chimera-B constructs were consistently more active than their respective fully synthetic L1 constructs (Table 1, rows 4 & 5). The B chimeras consisted of the fully synthetic ORF1 and the first three quarters of synthetic ORF2, with the last quarter of ORF2 derived from the native L1_RP _element.

Similar trends were observed when a two-step retrotransposition assay [1] was used, in which the cells underwent selection for a *Neo-AI *reporter, after enrichment for a population of plasmid-bearing cells using puromycin selection (see Additional file 2. Table S2), indicating that these effects are dependent on intrinsic aspects of retrotransposition, not on the specific retrotransposition reporter used.

### The 3' UTR, inter-ORF and constitutive transport elements are not essential to ORFeus-Hs retrotransposition

We noted that several of the initial synthetic element constructs we made had extremely short 3' UTRs. A portion of the 3' UTR is dispensable for (native) human L1 retrotransposition [1], thus we investigated whether the virtually complete absence of the UTR in these constructs could explain the modest increase in transposition rates (relative to the mouse synthetic elements). To test this theory, we made several constructs in which the full-length 3' UTR sequence was restored in the various *ORFeus*-Hs constructs, and tested their retrotransposition levels (see Additional file 2, Table S2). This alteration did increase the *ORFeus*-Hs retrotransposition frequency, but only by 1.1 to 2 times, compared with the construct without a 3' UTR (Table 2, rows 2 and 3).

**Table 2:** The 3' untranslated region and constitutive transport element are not essential to *ORFeus*-Hs retrotransposition.

Construct^a^	CMV^b^+5" UTR^c^	CMV+5" UTR+Kozak	CMV+Kozak
Fully native	1.0 ± 0.1 [pWA192]	ND^d^	ND
*ORFeus*-Hs, no 3" UTR	1.7 ± 0.4 [pWA180]	1.6 ± 0.5 [pWA181]	1.9 ± 0.4 [pWA200]
ORFeus-Hs + 3(UTR	2.4 ± 0.7 [pWA184]	2.8 ± 1.2 [pWA185]	2.0 ± 0.7 [pWA182]
*ORFeus*-Hs +3" UTR + CTE^e^	1.1 ± 0.4 [pWA199]	1.1 ± 0.6 [pWA186]	1.3 ± 0.2 [pWA183]

^a^For each construct, retrotransposition frequency is Retrotransposition frequencies are represented as fold changes relative to native L1_RP _[pWA192]. The average number of G418R colonies wass 157 per 104 cells seeded. Data are the mean ± SE of six independent experiments. Plasmid names are in square brackets.

^b^CMV = cytomegalovirus

^c^UTR = untranslated region

^d^ND = not determined.

^e^CTE = constitutive transport element

It has also been reported that the last 20 nucleotides of ORF2 and the first 70 nucleotides of the 3' UTR contain a constitutive transport element (CTE) that is important for the export of full-length mRNA to the cytoplasm [20]. Because this sequence is imbedded within ORF2, which was extensively recoded in *ORFeus*-Hs, it was a candidate to explain the difference in activity between chimera B and *ORFeus*-Hs. We evaluated the effect of restoring the CTE sequence alone to the wild-type sequence (synthetic sequence → native sequence). We therefore constructed plasmids in which both the native CTE and 3' UTR were restored to *ORFeus*-Hs. Interestingly, this maneuver actually reduced retrotransposition slightly (Table 2, rows 2 and 4), suggesting the presence of a sequence in the native CTE that is slightly inhibitory to retrotransposition by these constructs. It is important to recognize that the constructs described here contain introns, and thus they may be able to exit the nucleus via a mechanism distinct from that used by native L1 sequences, which do not normally undergo splicing as part of the retrotransposition process. Thus, it is formally possible that retrotransposition in the absence of an intron-containing reporter would actually depend on these sequences.

Finally, we replaced the native L1 inter-ORF region in *ORFeus*-Hs with a randomized version (see Methods). This randomization modestly increased the retrotransposition frequency of ORFeus (see Additional file 2, Table S2). Thus, as has been previously reported for native L1 [21,22], the native inter-ORF region sequence is also not crucial for retrotransposition of *ORFeus*-Hs.

### Synthetic sequence increases levels of L1 mRNA

**Figure 2:**
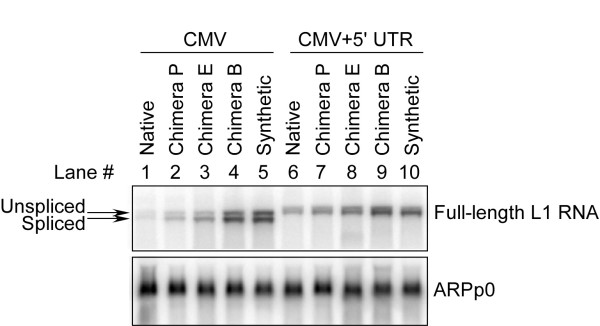
**Total RNA analysis of L1 expression**. Expression levels of native, partially synthetic, and completely synthetic *ORFeus*-Hs were compared in 293T cells. The vectors used were: pLD223, pWA172, pWA170, pWA176, pWA163, pWA174, pLD224, pLD227, pLD225, pWA165. Top, L1 mRNA expression; note both spliced and unspliced transcripts. Bottom, RNA expression of loading control, ARPP0.

**Figure 3:**
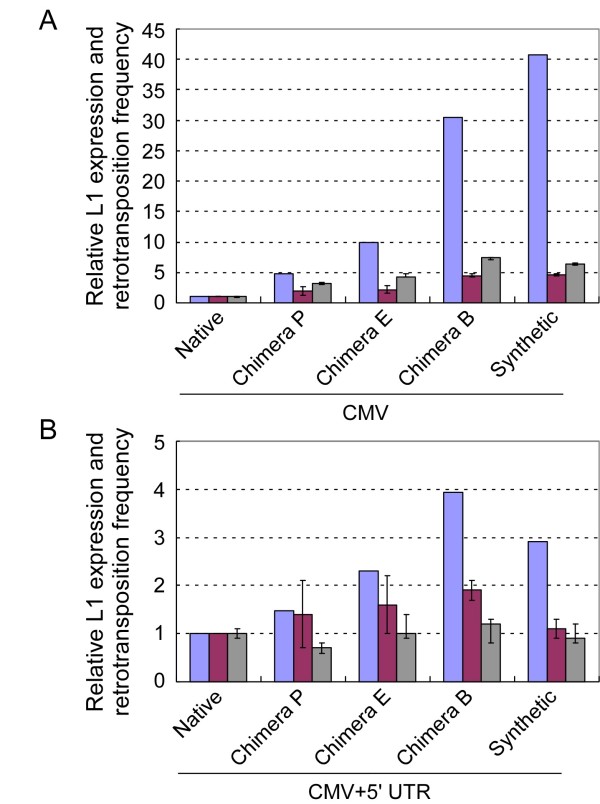
**Relative increases in RNA, protein and retrotransposition frequency. (A)** Relative increases in RNA, protein and retrotransposition frequency in the constructs containing CMV only. Chimera B = pWA176; chimera E = pWA170; chimera P = pWA172; native = pLD223; synthetic = pWA163. **(B**) Relative increases in RNA, protein and retrotransposition frequency in the constructs containing both CMV and 5' UTR promoters. Chimera E = pLD227; chimera B = pLD225; chimera P = pLD224; native = pWA174; synthetic = pWA165. Values of pLD223 and pWA174 were assigned as control in each group of constructs. Data are mean of a minimum of three independent experiments plus standard error. Blue = RNA; gray = relative retrotransposition frequency; purple = ORF1p.

We examined differences in transcript levels between the native L1_RP _constructs, *ORFeus*-Hs and the various semi-synthetic L1 chimeras. HEK-293T cells were transfected with the following vectors, all of which contain the CMV promoter: native L1_RP _with (pWA174) and without (pLD223) a L1 5' UTR sequence; the three chimeras with increasing lengths of synthetic sequence with (pLD224, pLD227, pLD225) and without (pWA172, pWA170, pWA176) L1 5' UTR sequences; and the fully synthetic *ORFeus*-Hs constructs with (pWA165) and without (pWA163) L1 5' UTRs. The cells were harvested at 24 hours post-transfection and total RNA was isolated. Levels of EGFP-containing transcripts were measured by RNA blotting and normalized to control (acidic ribosomal phosphoprotein P0; ARPP0) transcript levels (Figure 2). Lanes 1 to 5 show constructs without a 5' UTR and lanes 6 to 10 show those with a 5' UTR. Spliced and unspliced L1 mRNAs can be identified by their predicted mobilities in both cases. In all cases, mRNA levels increased monotonically, but not linearly, with retrotransposition levels. Amounts of the *ORFeus*-Hs L1 transcripts (Figure 2, lanes 5 and 10) were significantly greater than those observed in their native counterparts (Figure 2, lanes 1 and 6). As the length of the synthetic segment in the chimeric constructs increased, so did L1 transcript levels (Figure 2, lanes 2 to 4 and 7 to 9). In addition, chimera B constructs appeared to have slightly higher transcript levels than did the fully synthetic *ORFeus*-Hs constructs (Figure 2, lanes 9 and 10). Finally, we observed a complex context effect: the first three constructs (native, chimera P and chimera E), which had both CMV and L1 5' UTR promoters, had higher transcript levels than those with a CMV promoter alone. This may account for the higher retrotransposition levels observed. By contrast, the last two plasmids (chimera B and fully synthetic) showed the opposite effect, with the CMV promoter alone producing more RNA than the combination of CMV and 5' UTR (Figure 3).

### Increases in ORF1p expression and ORF2 RT activity in ORFeus-Hs

**Figure 4:**
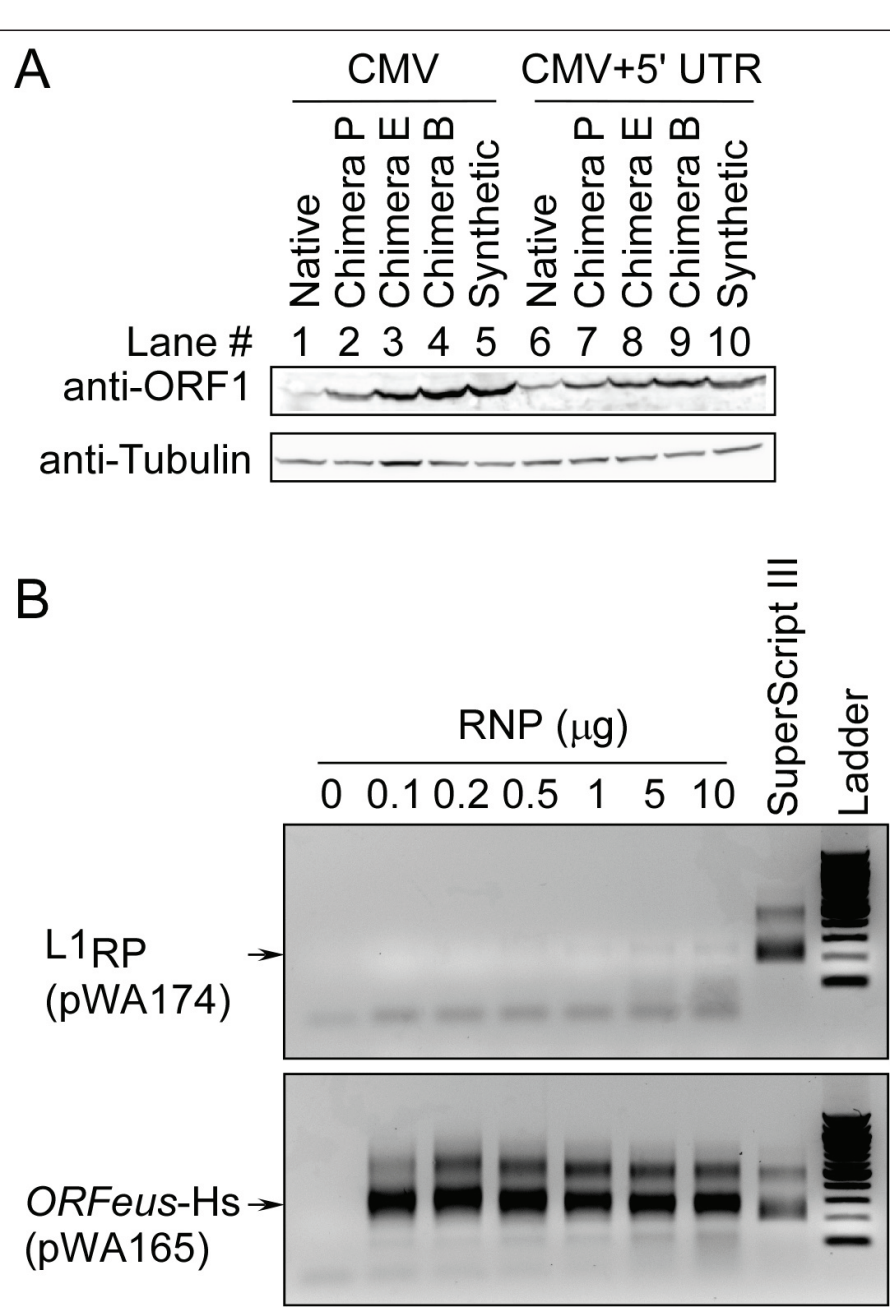
**Analysis of ORF1 protein expression and relative ORF2 RT activity**. **(A) **The same constructs as in Figure 2 were analyzed. The vectors used were: pLD223, pWA172, pWA170, pWA176, pWA163, pWA174, pLD224, pLD227, pLD225, pWA165. Top, protein expression of ORF1. Bottom, protein expression of the tubulin loading control. **(B) **L1 element amplification protocol (LEAP): assay was performed using ribonucleoproteins (RNPs) prepared from cells transfected with pWA174 (L1_RP_) and pWA165 (*ORFeus*-Hs). The numbers 0, 0.1, 0.2, 0.5, 1, 5 and 10 indicate the amount (μg) of total protein of the RNP prep added to each LEAP reaction. The arrows indicate the mobility of LEAP PCR product. SuperScript III represents a positive control in which 100 U of SuperScript III reverse transcriptase (Invitrogen, Carlsbad, CA, USA) was added to the LEAP reaction.

To determine the effect of our codon optimization on the levels of protein expression, HEK-293T cells were transfected and harvested as described above. After lysis, cells were analyzed by SDS-PAGE, transferred to membranes, and probed with anti-ORF1p antibody (Figure 4A). Similar to the levels of RNA transcripts, levels of ORF1 protein were considerably elevated for the synthetic and partly synthetic L1s (Figure 4A). The results were quantified by densitometry (Figure 3). In cells transfected with the native L1_RP _constructs (pLD223, pWA174), only a low level of ORF1p was observed at ~41 kDa (Figure 4A, lanes 1 and 6). As with RNA, the levels of ORF1p increased as the length of synthetic sequence was increased, but the extent of the increase in ORF1 protein was much less impressive (Figure 4A, lanes 2 to 5 and 7 to 10). The RNA, protein and retrotransposition increases correlated in terms of whether retrotransposition increased or decreased in each construct. Notably, levels of ORF1p from the chimera-B L1 elements (pWA176 and pLD225) were also slightly higher than those observed in the fully synthetic *ORFeus*-Hs elements (pWA163, pWA165) (Figure 3, Figure 4A).

Immunoblotting for ORF2 protein was not of sufficient quality to allow quantification. Instead, to evaluate ORF2p activity from *ORFeus*-Hs, we performed an L1 element amplification protocol (LEAP) [23] using ribonucleoprotein (RNP) prepared from 293T cells transfected with either L1_RP _(pWA174) or *ORFeus*-Hs (pWA165). As little as 0.1 μg RNP prepared from cells transfected with *ORFeus*-Hs produced a signal of strength equal to that produced by 10 μg native RNP (Figure 4B), but native L1 RNP did not produce a visible signal until at least 5 μg RNP was added. To roughly quantify the reverse transcription (RT) activity in these two samples, we titrated the *ORFeus*-Hs RNP down to 0.025 μg per reaction (see Additional file 1, Figure S3) and compared its activity with that of native L1 RNP. Approximately 10 μg native L1 RNP contained similar RT activity to that of ~0.05 μg *ORFeus*-Hs RNP (see Additional file 1: Figure S3B). Although this experiment is only semiquantitative, it is obvious that cells transfected with *ORFeus*-Hs displayed ORF2 RT activity of at least two orders of magnitude higher than those transfected with native L1. This activity increase is much more in line with RNA abundance than ORF1p abundance (see below), and suggests that L1 RNA may be limiting for RNP activity.

An interesting finding was that although the fold increases of L1 RNA and protein were all in the same direction, the magnitude of the increases was dramatically different (Figure 3). Comparing RNA increases with protein increases, it can readily be seen that in the chimera B and fully synthetic cases, the increases in RNA were much larger than the increases in ORF1 protein, by a factor of four to five in the CMV-only constructs. The retrotransposition frequency increases did not correlate well with RNA abundance in terms of fold increase, but were consistently 1.3 to 2 times larger in magnitude than the protein increases in the CMV promoter constructs. By contrast, for the CMV/L1 5' UTR promoter constructs, there was a larger increase in protein level than in retrotransposition frequency.

## Discussion

The data presented here provide an interesting contrast between the synthetic versions of human and mouse retrotransposons, *ORFeus*-Hs and *ORFeus*-Mm. Our previous data showed that the generation of *ORFeus*-Mm with optimized codons, which were presumably free of sequences that might hamper transcription, resulted in a highly active element with levels of retrotransposition that were as much as 200-fold higher than the native element [16]. These were shown to be in part due to higher levels of mouse L1 transcripts [15], and presumably correspondingly higher levels of protein products. However, when similar techniques were attempted in order to develop a highly active human retrotransposon, we were only able to increase levels of retrotransposition by a maximum of two to three times. Contrary to the findings in mouse L1 elements, the synthetic sequences did not increase human L1 protein and retrotransposition levels by the same margin.

Native L1 elements contain premature polyadenylation sites [15,24] and cryptic splice sites [25] that produce premature polyadenylated and spliced form L1 RNAs. These isoform RNAs could limit full-length L1 RNA production or compete for L1-encoded ORFs [25]. In our recoding process, most or all of these signals were removed, and this probably contributed to the increased abundance of full-length L1 RNA (Figure 2, Figure 3). Although the function of these signals in nature remains unknown, they are dispensable for L1 retrotransposition in tissue-culture assays.

One obvious reason that *ORFeus*-Hs did not increase retrotransposition frequency by 200 times is that the native mouse L1 element (L1_spa_) has much lower activity than the native human L1_RP _element [18]. In fact, codon optimization of both mouse and human L1 elements increased their retrotransposition abilities to a similar level (Table 1). This could represent an upper limit of L1 retrotransposition that can be readily tolerated by tissue-culture cells and/or a rate-limiting step(s) during the process of retrotransposition. Elevated levels of L1 RNA/protein and shuttling between nucleus and cytoplasm may have a strong effect on the cell, perhaps overloading its full capacity to process L1-RNP retrotransposition intermediates [26]. Consistent with this, we observed that cells overexpressing either *ORFeus*-Mm or *ORFeus*-Hs displayed considerably higher sensitivity to antibiotics than those transfected with native L1s. For example, HEK-293T cells transfected with *ORFeus*-Hs grew more slowly at a concentration of 2 μg/ml puromycin than did cells overexpressing native L1_RP _(see Additional file 1, Figure S4). These results are consistent with studies that reported effects on L1 protein expression leading to high levels of double-strand breaks and/or apoptosis and/or cellular senescence [27-30]. It is formally possible that codon optimization changes made in *ORFeus *corrected a mutation(s) in a *cis *element(s) that hampers retrotransposition efficiency of native L1, but results from both the study of Han *et al*. [16] on the mouse element and the present study on the human element show that L1 activity increases progressively as larger proportions of the native sequence are recoded, consistent with the reported elongation defect.

We noted that the three sets of L1 constructs driven by CMV only, CMV plus 5' UTR or the 5' UTR only had very different trends of retrotransposition frequency changes. This suggests that different promoters somehow produce RNAs that are of different 'quality'. One difference in quality is the structure of the RNA 5' end; the CMV promoter fragment also contains the 51 bp viral 5' UTR upstream of the L1 ORF1 AUG motif, whereas the native L1 promoter introduces the native 907 bp L1 5' UTR in its place. In the double-promoter construct, there are thought to be two transcription start sites, one identical to the native site, and one extended on its 5' end by the CMV 5' UTR. We have not directly examined the relative abundance of these two RNA forms. Thus, the 5' UTR sequences differ between the three types of elements, and it is possible that the interactions between the 5' UTRs and the rest of the RNA sequence influence retrotransposition efficiency.

Unexpected discrepancies in the increases in RNA abundance, protein abundance and retrotransposition frequency were noted between the various constructs, with RNA increasing much more dramatically than protein, and protein increasing more than retrotransposition frequency. This suggests that when comparing native versus synthetic RNA sequences, the latter either interferes with translational efficiency, decreases stability of the encoded protein, or both. The larger relative increase in RNA suggests that the primary effect of the codon optimization was to improve levels of full-length L1 mRNA. Because codon optimization is predicted to increase translational efficiency, it is surprising that protein levels actually decreased relative to RNA template abundance. It is formally possible that recoding leads to enhanced protein degradation. Native codon usage may provide signals for proper folding of the nascent protein [31]. Alternatively, if the interaction between the RNA and the protein to form RNP intermediates is abrogated in the fully or mostly synthetic elements, any ORF1 protein that does not get incorporated into RNPs may become very unstable, potentially explaining the RNA-protein discrepancy observed here.

Perhaps most surprisingly, we found that changes in ORF2 sequences in chimera B had a significant effect on the expression of ORF1p (compare pLD225 and pLD165 in Figure 4). This is consistent with models in which ORF2 protein, or the RNA sequence encoding it, might participate in the regulation of ORF1 protein translation or stability. Finally, although recoding of the human element did not produce an increase in retrotransposition frequency that was as dramatic as with *ORFeus*-Mm, *ORFeus*-Hs and its chimeric derivatives remain useful tools for L1 studies, and have the highest retrotransposition frequency of the available human L1s reported. Higher levels of full-length L1 mRNA and ORF1p provide a convenient and rapid marker for the early stages of the L1 replication cycle by various methods such as immunofluorescence and immunoprecipitation.

## Methods

### Plasmid construction

Synthetic human ORF1 and ORF2 sequences were created by replacing each codon in the human L1 ORFs with codons favored in strongly expressed human genes, and introducing strategically placed restriction enzyme sites using a DNA-shuffling approach [32]. Oligonucleotides (60-mer) (Integrated DNA Technologies, Coralville, IA, USA) collectively encoding both strands of *ORFeus-Hs *reading frames were used, and gene synthesis and assembly were performed as previously described [16]. Synthetic/native L1 chimeras were assembled by exploiting various native restriction sites (Figure 1; Additional file 2, Table S1). The sequence of the inter-ORF region was randomized by counting the number of each base in the native inter-ORF region. These were then randomized by selecting that number of each base, using the order of their occurrence in a famous novel [33] (Table 3).

**Table 3:** Sequences used for constructs.

Name	Sequence 5→'3'
Inter-ORF^a^	AATGGTTTTATACTCTAATCACTGCTAATTATTTCTTTTATTTGACTAGGCGCGCCCATCATA^b^

Primers for probes	

EGFP forward (JB13766)	ACGTAAACGGCCACAAGTTC

EGFP reverse (JB13767)	AAGTCGTGCTGCTTCATGTG

ARPP0 Forward	ACTGTGCCAGCCCAGAACAC

ARPP0 Reverse	GCAGATGGATCAGCCAAGAAG

LEAP^c^	

JB11560	GCGAGCACAGAATTAATACGACTCACTATAGGTTTTTTTTTTTT

JB11564	GCGAGCACAGAATTAATACGACTC

JB14067	GGATCCAGACATGATAAGATACATTGATGA

^a^Sequences between open reading frames.

^b^Sequence obtained by randomizing according to the occurrence of the letters in a famous novel (see Methods).

^c ^L1 element amplification protocol.

### Cell culture, transfection and retrotransposition assay

Retrotransposition assays with *EGFP-AI *indicators were performed in HEK-293T cells. HEK-293T cells (ATCC, Manassas, VA, USA) were maintained in Dulbecco modified Eagle medium (DMEM; Invitrogen, Carlsbad, CA, USA) with 10% fetal bovine serum (FBS) and penicillin/streptomycin (penicillin, 100 units/ml; streptomycin, 100 μg/ml), and cells were passaged upon confluence. HEK-293T cells were seeded at 2×10^5 ^cells per well in six-well plates and grown overnight. The next day, transfections were performed with 1 μg plasmid and 2.5 μl transfection reagent (Fugene HD; Roche Applied Science, Indianapolis, IN, USA) according to the manufacturer's protocol. The day after transfection, cells were treated with trypsin and transferred to 60 mm plates with complete medium containing puromycin 1 μg/ml. After 3 days of puromycin selection, cells were washed in 1× phosphate-buffered saline (PBS), and kept on ice before undergoing FACS (FACSCalibur instrument; BD Biosciences, Sparks, MD, USA), using forward scatter versus green fluorescence plots. The gating for EGFP-positive cells was determined by analyzing cells transfected with an expression plasmid (pCEP-Puro; puromycin-resistant, EGFP-negative). A minimum of 20,000 cells per sample were analyzed. Data were analyzed using CellQuest software. A minimum of eight independent experiments were performed for each construct.

Retrotransposition assays with *Neo-AI *indicators were performed with HeLa cells. HeLa cells were maintained in DMEM (Invitrogen, Carlsbad, CA, USA) supplemented with 10% FBS and penicillin/streptomycin, and were passaged upon confluence. Cells were seeded at 2.5×10^5 ^per well in a six-well plate, transfected (FuGene6; Roche Applied Science, Indianapolis, IN, USA) on the following day, and selected by growing with puromycin 2.5 μg/ml for 3 days. For each transfection, three 100 mm dishes were seeded with 1×10^4 ^to 4×10^4 ^cells each under G418 selection (500 μg/ml) for 10 to 14 days. A minimum of six independent experiments was performed for each construct. Retrotransposition activity was normalized to activity of L1_RP_.

### Northern blot assays

Total RNA was purified (RNeasy; Qiagen,Valencia, CA,USA), then 10 μg RNA was loaded on a 1.2% agarose/formaldehyde gel, blotted overnight to a nylon membrane (Genescreen plus; Perkin Elmer,Waltham, MA, USA) in 10× saline sodium citrate (SSC), crosslinked by ultraviolet radiation, and baked. Prehybridizations and hybridizations were both performed in an ultrasensitive hybridization buffer (ULTRAhyb; Applied Biosystems/Ambion, Austin, TX, USA) at 42°C. Washes were performed in 2×SSC with 0.1% sodium dodecyl sulfate (SDS) and in 0.1× SSC with 0.1% SDS. Radioactive signals were detected with a phosphoimager (Typhoon; GE Healthcare, Piscataway, NJ, USA) and quantified using ImageQuant software. Northern probes were first amplified by PCR, purified in gels [alpha 32P] labeled with TP (Random Prime-It II Kit, Stratagene, Santa Clara, CA, USA). Primers (Table 3) were used to amplify the EGFP and ARPP0 probes.

### Immunoblot assays

After the 3-day puromycin selection, the cells were lysed in buffer (M-PER; Thermo Scientific, Rockford, IL, USA) and spun in a centrifuge for 15 minutes at 13,000 g to separate out the cell lysate. The cell lysate (10 μl) was mixed with 10 μl 2× loading buffer (0.1 mol/l Tris-HCl pH 6.8, with 4.0% SDS, 20% glycerol, 5% β-mercaptoethanol and 0.2% bromophenol blue), and samples were separated in 4% to 20% SDS-PAGE gels. After transfer to nitrocellulose membranes, membranes were probed with anti-ORF1p IgY antibody, which had been generated by immunizing chickens with purified human L1 ORF1p overexpressed in *Escherichia coli *and purified from yolks (Gallus Immunotech Inc,Fergus, ON, Canada). Western blots were developed with detection reagent (ECL-plus; GE Healthcare, Piscataway, NJ, USA), detected using an imaging system (LAS3000 instrument (Fujifilm) and Image Reader LAS-3000 software) at 'high' setting. The signals in the electronic file were quantified using Multi-Gauge software (Fuji Film), a program based on band density. Only the nonsaturated signals were quantified, and the background was subtracted. Results were normalized by using the tubulin controls as a reference, and are presented as fold difference relative to fully native construct. A total of three western blots were performed, and a representative blot is shown.

### LEAP

LEAP was performed according to Kulpa *et al*. [23]. Briefly, 293T cells were transfected with pWA174 (L1RP) or pWA165 (*ORFeus*-Hs), and then selected on puromycin 1 μg/ml for 2 weeks. On harvest day, ~800 million cells were washed with PBS three times and then resuspended in 10 ml cold PBS. Cells were pelleted at 3,000 g for 5 minutes in a swinging bucket rotor, then lysed with 1 ml buffer (1.5 mmol/l KCl, 2.5 m mol/l MgCl_2_, 5 m mol/l Tris-HCl, 1% deoxycholic acid, 1% Triton X-100, 1× protease inhibitor cocktail) for 5 min on ice. The lysate was clarified by centrifugation at 3,000 g for 5 min at 4°C, and the supernatant was transferred to an 8.5% to 17% sucrose cushion. The gradient was spun at 39,000 rpm (SW40.1 rotor) (178,000 g) for 2 h at 4°C. The pellet was resuspended in 100 μL 5 mmol/l Tris (pH 7.5) with 1× protease inhibitor, and with glycerol added to give a final concentration of 50%. RNP preparations were stored at -80°C.

For the LEAP reaction, various amounts of RNP were added to 50 μl of 50 mmol/l Tris-HCl pH 7.5, 50 mmol/l KCl, 5 mmol/l MgCl_2_, 10 mmol/l dithiothreitol, 0.4 μmmol/l 3' LEAP primer (JB11560; Table 3), 20 units RNasin, 0.2 mmol/l dNTPs and 0.05% Tween 20, and incubated at 37°C for 1 hour. LEAP reaction product (1 μL) was used as template in a 50 μl PCR assay with 5 μl 10× PCR buffer (Roche Applied Science, Indianapolis, IN, USA), 5 μl 2.5 mmol/l dNTPs, 1 μl each of primers JB11564 (5 μmol/l)and JB14067 primers (5 μmol/l) (Table 3), and 1 μl FastStart *Taq *polymerase (Roche Applied Science, Indianapolis, IN, USA). The reaction was carried out at with an initial denaturation at 94°C for 5 min, followed by 35 cycles of 94°C for 30 seconds, 56°C for 30 seconds, and 72°C for 30 seconds, and final extension at 72°C for 7 minutes. An aliquot (10 μL) of each PCR product was loaded onto a 1.5% agarose gel. Band density was quantified using Multi-Gauge software.

### Cell viability assay

293T cells were transfected with pWA174 (L1_RP_), pLD225 (chimera B) or pWA165 (*ORFeus*-Hs) in six-well plates (2 μg L1 plasmid + 10 ng pCAG-eGFP/40000 cells). The following day, cells were treated with trypsin, and an equal number of cells were plated in a 96-well plate (~10,000 cells/well) with puromycin 2 μg/ml, or without puromycin selection. Another portion of the cells was analyzed by FACS to acquire transfection efficiency. Two days later, 20 μl cell-viability solution (CellTiter-Blue^®^; Promega, Madison, WI, USA) was added to each well. The plate was incubated at 37°C for 2 hours and then read, using a microplate reader with an excitation wavelength 550 nm and an emission wavelength of 600 nm. Relative cell viability is presented as the ratio of viable puromycin-resistant cells divided by total viable cells (without puromycin selection). Four independent transfections were performed for each construct, and all values were normalized to the transfection efficiency acquired by FACS.

## Competing interests

The authors declare that they have no competing interests.

## Authors' contributions

WA, LD, JSH and JDB designed the experiments, WA, LD, AMN, AY, KAO, and JSH performed the experiments. LD, AMN and JDB wrote the manuscript. All authors read and approved the final manuscript.

## Supplementary Material

Additional file 1 **Supplementary Figures 1-4**. **(1) **Alignment of native human L1_RP _with *ORFeus*-Hs. BioEdit was used to create a nucleic acid alignment of native human L1 and *ORFeus*-Hs, starting at the ATG of open reading frame (ORF)1 and ending at the stop codon of ORF2. For these sequences, the base composition of L1_RP _is 40% A (1998), 21% C (1047), 19% G (906), 20% T (967). The base composition of *ORFeus*-HS is 27% A (1322), 34% C (1648), 27% (1314) G, 12% T (624). L1_RP _(Genbank accession number AF148856) was used as the sequence for native human L1. Identities are marked with asterisks. Start and stop codons of ORF1 and ORF2, restriction sites used to clone building blocks (*Mfe*I, *BsmB*I, *Asc*I, *Age*I, *BstB*I, *Nru*I, *Xma*I, *Mlu*I, *Nhe*I, *EcoR*V, *Nde*I, *Cla*I, *Xho*I) and make native/synthetic chimeras (*Pml*I, *EcoR*I and *BamH*I) are highlighted in gray boxes. **(2) **Alignment of different promoters used in this study. Kozak sequence and boundaries of cytomegalovirus (CMV) and the 5' untranslated region (UTR) are highlighted. **(3) **Quantification of L1 element amplification protocol (LEAP). **(A) **LEAP was performed using a ribonucleoprotein (RNP) preparation with the indicated amount, and an equal amount of PCR product was loaded onto a 1.5% agarose gel. The arrow indicates the mobility of LEAP PCR product on the gel. **(B) **The density of the bands was quantified using the Multi-Gauge program and plotted as a function of the amount of *ORFeus*-Hs RNP. A trend line was drawn using values from lane 3 to lane 6 and the *X*-axis values from lane 1 to lane 2 (0.03 and 0.05 μg respectively) were calculated based on the trend line. Blue diamond = data from *ORFeus*-Hs RNP (lanes 3 to 8); red triangle = data from L1_RP _RNP (lanes 1 and 2). *X*-axis, amount of *ORFeus*-Hs RNP in uG; Y-axis, pixel value. **(4) **Cell-viability assay. Relative cell viability is presented as the ratio of viable puromycin-resistant cells divided by total viable cells (without puromycin selection). Four independent transfections were performed for each construct, and triplet reading was acquired from each transfection. All values were normalized to the transfection efficiency acquired by fluorescence-activated cell sorting, and standard error is shown. Click here for file

Additional file 2 **Supplementary Tables**. Tables 1 and 2. Click here for file
