# Supplementary material for: Characterization of a synthetic human LINE-1 retrotransposon ORFeus-Hs
Source: Mob DNA. 2011 Feb 14;2:2. doi: 10.1186/1759-8753-2-2 (PMC3045867; doi:10.1186/1759-8753-2-2)

Supplementary Figure 1

ORF1 start

```
                ....|....| ....|....| ....|....| ....|....| ....|....|
                        10         20         30         40         50
L1RP            ATGGGAAAAA AACAGAACAG AAAAACTGGA AACTCTAAAA CGCAGAGCGC
ORFeus-Hs       ATGGGCAAGA AGCAGAACCG CAAGACCGGC AACAGCAAGA CCCAGAGCGC
                ***** ** * * ****** *  ** ** **  ***   ** * * ********

                ....|....| ....|....| ....|....| ....|....| ....|....|
                        60         70         80         90        100
L1RP            CTCTCCTCCT CCAAAGGAAC GCAGTTCCTC ACCAGCAACA GAACAAAGCT
ORFeus-Hs       CAGCCCCCCC CCCAAGGAGC GCAGCAGCAG CCCCGCCACC GAGCAGAGCT
                *   ** **  ** ***** * ****   *    ** ** **  ** ** ****

                ....|....| ....|....| ....|....| ....|....| ....|....|
                       110        120        130        140        150
L1RP            GGATGGAGAA TGATTTTGAT GAGCTGAGAG AAGAAGGCTT CAGACGATCA
ORFeus-Hs       GGATGGAGAA CGACTTCGAC GAGCTGCGCG AGGAGGGCTT CCGCCGCAGC
                **********  ** ** **  ****** * * * ** ***** * * **

                ....|....| ....|....| ....|....| ....|....| ....|....|
                       160        170        180        190        200
L1RP            AATTACTCTG AGCTACGGGA GGACATTCAA ACCAAAGGCA AAGAAGTTGA
ORFeus-Hs       AACTACAGCG AGCTGCGCGA GGACATCCAG ACCAAGGGCA AGGAGGTGGA
                ** ***   * **** ** ** ****** **  ***** **** * ** ** **

                ....|....| ....|....| ....|....| ....|....| ....|....|
                       210        220        230        240        250
L1RP            AAACTTTGAA AAAAATTTAG AAGAATGTAT AACTAGAATA ACCAATACAG
ORFeus-Hs       GAACTTCGAG AAGAACCTGG AGGAGTGCAT CACCCGCATC ACCAACACCG
                 ***** **  ** **  * * * ** ** **  **  * **  ***** ** *

                ....|....| ....|....| ....|....| ....|....| ....|....|
                       260        270        280        290        300
L1RP            AGAAGTGCTT AAAGGAGCTG ATGGAGCTGA AAACCAAGGC TCGAGAACTA
ORFeus-Hs       AGAAGTGCCT GAAGGAGCTG ATGGAGCTGA AGACCAAGGC CCGCGAGCTG
                ******** *  ********* ********** * ********  ** ** **

                ....|....| ....|....| ....|....| ....|....| ....|....|
                       310        320        330        340        350
L1RP            CGTGAAGAAT GCAGAAGCCT CAGGAGCCGA TGCGATCAAC TGGAAGAAAG
ORFeus-Hs       CGCGAGGAGT GCCGCAGCCT GCGCAGCCGC TGCGACCAAT TGGAGGAGCG
                ** ** ** * ** * *****   * *****  ***** ***  **** **  *
                                                      Mfe I

                ....|....| ....|....| ....|....| ....|....| ....|....|
                       360        370        380        390        400
L1RP            GGTATCAGCA ATGGAAGATG AAATGAATGA AATGAAGCGA GAAGGGAAGT
ORFeus-Hs       CGTGAGCGCC ATGGAGGACG AGATGAACGA GATGAAGCGC GAGGGCAAGT
                 **    **  ***** ** * * ***** **  ********  ** ** ****

                ....|....| ....|....| ....|....| ....|....| ....|....|
                       410        420        430        440        450
L1RP            TTAGAGAAAA AAGAATAAAA AGAAATGAGC AAAGCCTCCA AGAAATATGG
ORFeus-Hs       TCCGCGAGAA GCGCATCAAG CGCAACGAGC AGAGCCTGCA GGAGATCTGG
                *  * ** **   * ** **   * ** **** * ***** **  ** ** ***
```

```
              ....|....| ....|....| ....|....| ....|....| ....|....|
                     460        470        480        490        500
L1RP          GACTATGTGA AAAGACCAAA TCTACGTCTG ATTGGTGTAC CTGAAAGTGA
ORFeus-Hs     GACTACGTGA AGCGCCCCAA CCTGCGCCTG ATCGGCGTGC CCGAGAGCGA
              ***** **** *  * ** **  ** ** *** ** ** ** * * ** ** **

              ....|....| ....|....| ....|....| ....|....| ....|....|
                     510        520        530        540        550
L1RP          TGTGGAGAAT GGAACCAAGT TGGAAAACAC TCTGCAGGAT ATTATCCAGG
ORFeus-Hs     CGTGGAGAAC GGCACCAAGC TGGAGAACAC CCTGCAGGAC ATCATCCAGG
               ********  ** ******  **** *****  ********  ** *******

              ....|....| ....|....| ....|....| ....|....| ....|....|
                     560        570        580        590        600
L1RP          AGAACTTCCC CAATCTAGCA AGGCAGGCCA ACGTTCAGAT TCAGGAAATA
ORFeus-Hs     AGAACTTCCC CAACCTGGCC CGCCAGGCCA ACGTGCAGAT CCAGGAGATC
              ********** *** ** **   * ******* **** *****  ***** **

              ....|....| ....|....| ....|....| ....|....| ....|....|
                     610        620        630        640        650
L1RP          CAGAGAACGC CACAAAGATA CTCCTCGAGA AGAGCAACTC CAAGACACAT
ORFeus-Hs     CAGCGCACCC CCCAGCGCTA CAGCAGCCGC CGCGCCACCC CCCGCCACAT
              *** * ** * * **  * ** *  *    *    * ** ** * *  * *****

              ....|....| ....|....| ....|....| ....|....| ....|....|
                     660        670        680        690        700
L1RP          AATTGTCAGA TTCACCAAAG TTGAAATGAA GGAAAAAATG TTAAGGGCAG
ORFeus-Hs     CATCGTGCGC TTCACCAAGG TGGAGATGAA GGAGAAGATG CTGCGCGCCG
               ** **  *  ******** * * ** ***** *** ** ***  *  * ** *

              ....|....| ....|....| ....|....| ....|....| ....|....|
                     710        720        730        740        750
L1RP          CCAGAGAGAA AGGTCGGGTT ACCCTCAAAG GAAAGCCCAT CAGACTAACA
ORFeus-Hs     CCCGCGAGAA GGGCCGCGTG ACCCTGAAGG GCAAGCCCAT CCGTCTCACC
              ** * *****  ** ** **  ***** ** * * ******** * * ** **
                                                               BsmB I
              ....|....| ....|....| ....|....| ....|....| ....|....|
                     760        770        780        790        800
L1RP          GCGGATCTCT CGGCAGAAAC CCTACAAGCC AGAAGAGAGT GGGGGCCAAT
ORFeus-Hs     GCCGACCTGA GCGCCGAGAC CCTGCAGGCC CGCCGCGAGT GGGGCCCCAT
              ** ** **      ** ** ** *** ** ***  *  * **** **** ** **

              ....|....| ....|....| ....|....| ....|....| ....|....|
                     810        820        830        840        850
L1RP          ATTCAACATT CTTAAAGAAA AGAATTTTCA ACCCAGAATT TCATATCCAG
ORFeus-Hs     CTTCAACATC CTGAAGGAGA AGAACTTCCA GCCCCGCATC AGCTACCCCG
               ********  ** ** ** * **** ** **  *** * **     ** ** *

              ....|....| ....|....| ....|....| ....|....| ....|....|
                     860        870        880        890        900
L1RP          CCAAACTAAG CTTCATAAGT GAAGGAGAAA TAAAATACTT TATAGACAAG
ORFeus-Hs     CCAAGCTGAG CTTCATCAGC GAGGGCGAGA TCAAGTACTT CATCGACAAG
              **** ** ** ****** **  ** ** ** * * ** *****  ** ******
```

```
              ....|....| ....|....| ....|....| ....|....| ....|....|
                     910        920        930        940        950
L1RP          CAAATGTTGA GAGATTTTGT CACCACCAGG CCTGCCCTAA AAGAGCTCCT
ORFeus-Hs     CAGATGCTGC GCGACTTCGT GACCACCCGC CCCGCCCTGA AGGAGCTGCT
              ** *** **  * ** ** **  ****** *  ** ***** * * ***** **

              ....|....| ....|....| ....|....| ....|....| ....|....|
                     960        970        980        990       1000
L1RP          GAAGGAAGCG CTAAACATGG AAAGGAACAA CCGGTACCAG CCGCTGCAAA
ORFeus-Hs     GAAGGAGGCC CTGAACATGG AGCGCAACAA CCGCTACCAG CCCCTGCAGA
              ****** **  ** ******* *  * ***** *** ****** ** ***** *

                    ORF1 stop
              ....|....| ....|....| ....|....| ....|....| ....|....|
                    1010       1020       1030       1040       1050
L1RP          ATCATGCCAA AATGTAAAGA CCATCAAGAC TAGGAAGAAA CTGCACCAAC
ORFeus-Hs     ACCACGCCAA GATGTAAAGA CCATCGAGAC TAGGAAGAAA CTGCATCAAC
              * ** *****  ********* ***** **** ********** ***** ****

                                   ORF2 start
              ....|....| ....|....| ....|....| ....|....| ....|....|
                    1060       1070       1080       1090       1100
L1RP          TAATGAGCAA AATCACCAGC TAACATCATA ATGACAGGAT CAACTTCACA
ORFeus-Hs     TAATGAGCAA AATCAGGCGC GCCCATCATA ATGACCGGCA GCAACAGCCA
              ********** *****   **    ******* ***** **     *     **
                            Asc I

              ....|....| ....|....| ....|....| ....|....| ....|....|
                    1110       1120       1130       1140       1150
L1RP          CATAACAATA TTAACTTTAA ATATAAATGG ACTAAATTCT GCAATTAAAA
ORFeus-Hs     CATCACCATC CTGACCCTGA ACATCAACGG CCTGAACAGC GCCATCAAGC
              *** ** **   * **  * * * ** ** **  ** **      ** ** **

              ....|....| ....|....| ....|....| ....|....| ....|....|
                    1160       1170       1180       1190       1200
L1RP          GACACAGACT GGCAAGTTGG ATAAAGAGTC AAGACCCATC AGTGTGCTGT
ORFeus-Hs     GCCACCGCCT GGCCAGCTGG ATCAAGAGCC AGGACCCCAG CGTGTGCTGC
              * *** * ** *** ** *** ** ***** * * *****     ********

              ....|....| ....|....| ....|....| ....|....| ....|....|
                    1210       1220       1230       1240       1250
L1RP          ATTCAGGAAA CCCATCTCAC GTGCAGAGAC ACACATAGGC TCAAAATAAA
ORFeus-Hs     ATCCAGGAGA CCCACCTCAC GTGCCGCGAC ACCCACCGCC TGAAGATCAA
              ** ***** * **** ***** **** * *** ** **  * * * ** ** **
                             Pml I

              ....|....| ....|....| ....|....| ....|....| ....|....|
                    1260       1270       1280       1290       1300
L1RP          AGGATGGAGG AAGATCTACC AAGCCAATGG AAAACAAAAA AAGGCAGGGG
ORFeus-Hs     GGGCTGGCGC AAGATCTACC AGGCCAACGG CAAGCAGAAG AAGGCCGGCG
               ** *** *  ********** * ***** **  ** ** **  ***** ** *

              ....|....| ....|....| ....|....| ....|....| ....|....|
                    1310       1320       1330       1340       1350
L1RP          TTGCAATCCT AGTCTCTGAT AAAACAGACT TTAAACCAAC AAAGATCAAA
ORFeus-Hs     TGGCCATCCT GGTGAGCGAC AAGACCGACT TCAAGCCCAC CAAGATCAAG
              * ** *****  **    **  ** ** **** * ** ** **  ********
```

```
            ....|....| ....|....| ....|....| ....|....| ....|....|
                   1360       1370       1380       1390       1400
L1RP        AGAGACAAAG AAGGCCATTA CATAATGGTA AAGGGATCAA TTCAACAAGA
ORFeus-Hs   CGCGACAAGG AGGGCCACTA CATCATGGTG AAGGGCAGCA TCCAGCAGGA
             * ***** * * ***** ** *** *****  *****    * * ** ** **

            ....|....| ....|....| ....|....| ....|....| ....|....|
                   1410       1420       1430       1440       1450
L1RP        GGAGCTAACT ATCCTAAATA TTTATGCACC CAATACAGGA GCACCCAGAT
ORFeus-Hs   GGAGCTGACC ATCCTGAACA TCTACGCCCC CAACACCGGT GCCCCCCGCT
            ****** **  ***** ** * * ** ** ** *** ** **  ** *** * *
                                               Age I

            ....|....| ....|....| ....|....| ....|....| ....|....|
                   1460       1470       1480       1490       1500
L1RP        TCATAAAGCA AGTCCTCAGT GACCTACAAA GAGACTTAGA CTCCCACACA
ORFeus-Hs   TCATCAAGCA GGTGCTGAGC GACCTGCAGC GCGACCTGGA CAGCCACACC
            **** *****  ** ** **  ***** **   * *** * ** *  ******

            ....|....| ....|....| ....|....| ....|....| ....|....|
                   1510       1520       1530       1540       1550
L1RP        TTAATAATGG GAGACTTTAA CACCCCACTG TCAACATTAG ACAGATCAAC
ORFeus-Hs   CTGATCATGG GCGACTTCAA CACCCCCCTG AGCACCCTGG ACCGCAGCAC
             * ** **** * ***** ** ****** ***    **  * * ** *    **

            ....|....| ....|....| ....|....| ....|....| ....|....|
                   1560       1570       1580       1590       1600
L1RP        GAGACAGAAA GTCAACAAGG ATACCCAGGA ATTGAACTCA GCTCTGCACC
ORFeus-Hs   CCGCCAGAAG GTGAACAAGG ACACCCAGGA GCTGAACAGC GCCCTGCACC
              * *****  ** ******* * ********   *****    ** *******

            ....|....| ....|....| ....|....| ....|....| ....|....|
                   1610       1620       1630       1640       1650
L1RP        AAGCAGACCT AATAGACATC TACAGAACTC TCCACCCCAA ATCAACAGAA
ORFeus-Hs   AGGCCGACCT GATCGACATC TACCGCACCC TGCACCCCAA GAGCACCGAG
            * ** *****  ** ****** *** * ** * * ********     ** **

            ....|....| ....|....| ....|....| ....|....| ....|....|
                   1660       1670       1680       1690       1700
L1RP        TATACGTTTT TTTCAGCACC ACACCACACC TATTCCAAAA TTGACCACAT
ORFeus-Hs   TACACCTTCT TCAGCGCCCC CCACCACACC TACAGCAAGA TCGACCACAT
            ** ** ** * *    ** **  ********* **   *** * * ********

            ....|....| ....|....| ....|....| ....|....| ....|....|
                   1710       1720       1730       1740       1750
L1RP        AGTTGGAAGC AAAGCTCTCC TCAGCAAATG TAAAAGAACA GAAATTATAA
ORFeus-Hs   CGTGGGCAGC AAGGCCCTGC TGAGCAAGTG CAAGCGCACC GAGATCATCA
             ** ** *** ** ** ** * * ***** **  **  * **  ** ** ** *

            ....|....| ....|....| ....|....| ....|....| ....|....|
                   1760       1770       1780       1790       1800
L1RP        CAAACTATCT CTCAGACCAC AGTGCAATCA AACTAGAGCT CAGGATTAAG
ORFeus-Hs   CCAACTACCT GAGCGACCAC AGCGCCATCA AGCTGGAGCT GCGCATCAAG
            * ***** **      ****** ** ** **** * ** *****   * ** ***
```

```
              ....|....| ....|....| ....|....| ....|....| ....|....|
                      1810       1820       1830       1840       1850
L1RP          AATCTCACTC AAAGCCGCTC AACTACATGG AAACTGAACA ACCTGCTCCT
ORFeus-Hs     AACCTGACCC AGAGCCGCAG CACCACCTGG AAGCTGAACA ACCTGCTGCT
              ** ** ** * * ******    ** ** *** ** ******* ******* **

              ....|....| ....|....| ....|....| ....|....| ....|....|
                      1860       1870       1880       1890       1900
L1RP          GAATGACTAC TGGGTACATA ACGAAATGAA GGCAGAAATA AAGATGTTCT
ORFeus-Hs     GAACGACTAC TGGGTGCACA ACGAGATGAA GGCCGAGATC AAGATGTTCT
              *** ****** ***** ** * **** ***** *** ** **  **********

              ....|....| ....|....| ....|....| ....|....| ....|....|
                      1910       1920       1930       1940       1950
L1RP          TTGAAACCAA CGAGAACAAA GACACCACAT ACCAGAATCT CTGGGACGCA
ORFeus-Hs     TCGAAACCAA CGAGAACAAG GACACCACCT ACCAGAACCT GTGGGACGCC
              * ******** *********  ******** * ******* **  ********
              Bst BI

              ....|....| ....|....| ....|....| ....|....| ....|....|
                      1960       1970       1980       1990       2000
L1RP          TTCAAAGCAG TATGTAGAGG GAAATTTATA GCACTAAATG CCTACAAGAG
ORFeus-Hs     TTCAAGGCCG TGTGCCGCGG CAAGTTCATC GCCCTGAACG CCTACAAGCG
              ***** ** * * **  * **  ** ** **  ** ** ** * ******** *

              ....|....| ....|....| ....|....| ....|....| ....|....|
                      2010       2020       2030       2040       2050
L1RP          AAAGCAGGAA AGATCCAAAA TTGACACCCT AACATCACAA TTAAAAGAAC
ORFeus-Hs     CAAGCAGGAG CGCAGCAAGA TCGACACCCT GACCAGCCAG CTGAAGGAGC
               ********   *   *** * * ********  **     **    * ** ** *

              ....|....| ....|....| ....|....| ....|....| ....|....|
                      2060       2070       2080       2090       2100
L1RP          TAGAAAAGCA AGAGCAAACA CATTCAAAAG CTAGCAGAAG GCAAGAAATA
ORFeus-Hs     TGGAGAAGCA GGAGCAGACC CACAGCAAGG CCAGCCGCCG CCAGGAGATC
              * ** *****  ***** **  **    ** * * *** *  *  ** ** **

              ....|....| ....|....| ....|....| ....|....| ....|....|
                      2110       2120       2130       2140       2150
L1RP          ACTAAAATCA GAGCAGAACT GAAGGAAATA GAGACACAAA AAACCCTTCA
ORFeus-Hs     ACCAAGATCC GCGCCGAGCT GAAGGAGATC GAGACCCAGA AGACCCTGCA
              ** ** ***  * ** ** ** ****** **  ***** ** * * ***** **

              ....|....| ....|....| ....|....| ....|....| ....|....|
                      2160       2170       2180       2190       2200
L1RP          AAAAATCAAT GAATCCAGGA GCTGGTTTTT TGAAAGGATC AACAAAATTG
ORFeus-Hs     GAAGATCAAC GAGTCGCGAA GCTGGTTCTT CGAGCGCATC AACAAGATCG
               ** *****  ** **  * * ******* **  **  * *** ***** ** *
                              Nru I

              ....|....| ....|....| ....|....| ....|....| ....|....|
                      2210       2220       2230       2240       2250
L1RP          ATAGACCGCT AGCAAGACTA ATAAAGAAAA AAAGAGAGAA GAATCAAATA
ORFeus-Hs     ACCGCCCCCT GGCCCGCCTG ATCAAGAAGA AGCGCGAGAA GAACCAGATC
              *  * ** **  **  * **  ** ***** * *  * ***** *** ** **
```

```
           ....|....| ....|....| ....|....| ....|....| ....|....|
                  2260       2270       2280       2290       2300
L1RP       GACACAATAA AAAATGATAA AGGGGATATC ACCACCGATC CCACAGAAAT
ORFeus-Hs  GACACCATCA AGAACGACAA GGGCGACATC ACCACCGACC CCACCGAGAT
           ***** ** * * ** ** **  ** ** *** ******** * **** ** **

           ....|....| ....|....| ....|....| ....|....| ....|....|
                  2310       2320       2330       2340       2350
L1RP       ACAAACTACC ATCAGAGAAT ACTACAAACA CCTCTACGCA AATAAACTAG
ORFeus-Hs  CCAGACCACC ATCCGCGAGT ACTACAAGCA CCTGTACGCC AACAAGCTGG
            ** ** *** *** * ** * ******* ** *** *****  ** ** ** *

           ....|....| ....|....| ....|....| ....|....| ....|....|
                  2360       2370       2380       2390       2400
L1RP       AAAATCTAGA AGAAATGGAT ACATTCCTCG ACACATACAC TCTCCCAAGA
ORFeus-Hs  AGAACCTGGA GGAGATGGAC ACCTTCCTGG ACACCTACAC CCTGCCCCGC
           * ** ** **  ** *****  ** ***** * **** *****  ** **  *

           ....|....| ....|....| ....|....| ....|....| ....|....|
                  2410       2420       2430       2440       2450
L1RP       CTAAACCAGG AAGAAGTTGA ATCTCTGAAT CGACCAATAA CAGGCTCTGA
ORFeus-Hs  CTGAACCAGG AGGAGGTGGA GAGCCTGAAC CGCCCCATCA CCGGCAGCGA
           ** ******* * ** ** **     *****  ** ** ** * * ***   **

           ....|....| ....|....| ....|....| ....|....| ....|....|
                  2460       2470       2480       2490       2500
L1RP       AATTGTGGCA ATAATCAATA GTTTACCAAC CAAAAAGAGT CCAGGACCAG
ORFeus-Hs  GATCGTGGCC ATCATCAACA GCCTGCCCAC CAAGAAGAGC CCCGGCCCCG
            ** *****  ** ***** * *  * ** ** *** *****  ** ** ** *

           ....|....| ....|....| ....|....| ....|....| ....|....|
                  2510       2520       2530       2540       2550
L1RP       ATGGATTCAC AGCCGAATTC TACCAGAGGT ACAAGGAGGA ACTGGTACCA
ORFeus-Hs  ACGGCTTCAC CGCCGAATTC TACCAGCGCT ACAAGGAGGA GCTGGTGCCC
           * ** *****  ********* ****** * * **********  ***** **
                           EcoR I

           ....|....| ....|....| ....|....| ....|....| ....|....|
                  2560       2570       2580       2590       2600
L1RP       TTCCTTCTGA AACTATTCCA ATCAATAGAA AAAGAGGGAA TCCTCCCTAA
ORFeus-Hs  TTCCTGCTGA AGCTGTTCCA GAGCATCGAG AAGGAGGGCA TCCTGCCCAA
           ***** **** * ** *****     ** **  ** ***** * **** ** **

           ....|....| ....|....| ....|....| ....|....| ....|....|
                  2610       2620       2630       2640       2650
L1RP       CTCATTTTAT GAGGCCAGCA TCATTCTGAT ACCAAAGCCG GGCAGAGACA
ORFeus-Hs  CAGCTTCTAC GAGGCCAGCA TCATCCTGAT CCCCAAGCCC GGCCGCGACA
           *   ** ** ********** **** *****  ** *****  *** * ****

           ....|....| ....|....| ....|....| ....|....| ....|....|
                  2660       2670       2680       2690       2700
L1RP       CAACCAAAAA AGAGAATTTT AGACCAATAT CCTTGATGAA CATTGATGCA
ORFeus-Hs  CCACCAAGAA GGAGAACTTC CGCCCCATCA GCCTGATGAA CATCGACGCC
           * ***** **  ***** **   * ** **    * ******* *** ** **
```

```
                ....|....| ....|....| ....|....| ....|....| ....|....|
                      2710       2720       2730       2740       2750
L1RP            AAAATCCTCA ATAAAATACT GGCAAACCGA ATCCAGCAGC ACATCAAAAA
ORFeus-Hs       AAGATCCTGA ACAAGATCCT GGCCAACCGC ATCCAGCAGC ACATCAAGAA
                ** ***** * * ** ** ** *** *****  ********** ******* **

                ....|....| ....|....| ....|....| ....|....| ....|....|
                      2760       2770       2780       2790       2800
L1RP            GCTTATCCAC CATGATCAAG TGGGCTTCAT CCCTGGGATG CAAGGCTGGT
ORFeus-Hs       GCTGATCCAC CACGACCAGG TGGGCTTCAT CCCCGGGATG CAGGGCTGGT
                *** ****** ** ** ** * ********** *** ****** ** *******
                                                    Xma I

                ....|....| ....|....| ....|....| ....|....| ....|....|
                      2810       2820       2830       2840       2850
L1RP            TCAATATACG CAAATCAATA AATGTAATCC AGCATATAAA CAGAGCCAAA
ORFeus-Hs       TCAACATCCG CAAGAGCATC AACGTGATCC AGCACATCAA CCGCGCCAAG
                **** ** ** ***    **  ** ** **** **** ** ** * * *****

                ....|....| ....|....| ....|....| ....|....| ....|....|
                      2860       2870       2880       2890       2900
L1RP            GACAAAAACC ACATGATTAT CTCAATAGAT GCAGAAAAAG CCTTTGACAA
ORFeus-Hs       GACAAGAACC ACATGATCAT CAGCATCGAC GCCGAGAAGG CCTTCGACAA
                ***** **** ******* ** *   ** **  ** ** ** * **** *****

                ....|....| ....|....| ....|....| ....|....| ....|....|
                      2910       2920       2930       2940       2950
L1RP            AATTCAACAA CCCTTCATGC TAAAAACTCT CAATAAATTA GGTATTGATG
ORFeus-Hs       GATCCAGCAG CCCTTCATGC TGAAGACCCT GAACAAGCTG GGCATCGACG
                 ** ** **  ********** * ** ** **  ** **  *  ** ** ** *

                ....|....| ....|....| ....|....| ....|....| ....|....|
                      2960       2970       2980       2990       3000
L1RP            GGACGTATTT CAAAATAATA AGAGCTATCT ATGACAAACC CACAGCCAAT
ORFeus-Hs       GCACCTACTT CAAGATCATC CGCGCCATCT ACGACAAGCC CACCGCCAAC
                * ** ** ** *** ** **   * ** **** * ***** ** *** *****

                ....|....| ....|....| ....|....| ....|....| ....|....|
                      3010       3020       3030       3040       3050
L1RP            ATCATACTGA ATGGGCAAAA ACTGGAAGCA TTCCCTTTGA AAACCGGCAC
ORFeus-Hs       ATCATCCTGA ACGGCCAGAA GCTGGAGGCC TTCCCCCTGA AGACCGGCAC
                ***** **** * ** ** **  ***** **  *****  *** * ********

                ....|....| ....|....| ....|....| ....|....| ....|....|
                      3060       3070       3080       3090       3100
L1RP            AAGACAGGGA TGCCCTCTCT CACCGCTCCT ATTCAACATA GTGTTGGAAG
ORFeus-Hs       GCGTCAGGGC TGCCCCCTGA GCCCCCTGCT GTTCAACATC GTGCTGGAGG
                  * *****  ***** **     ** ** **  ********  *** **** *
                Mlu I

                ....|....| ....|....| ....|....| ....|....| ....|....|
                      3110       3120       3130       3140       3150
L1RP            TTCTGGCCAG GGCAATCAGG CAGGAGAAGG AAATAAAGGG TATTCAATTA
ORFeus-Hs       TGCTGGCCCG CGCCATCCGC CAGGAGAAGG AGATCAAGGG CATCCAGCTG
                * ****** *  ** *** *  ********** * ** *****  ** **  *
```

```
                ....|....| ....|....| ....|....| ....|....| ....|....|
                      3160       3170       3180       3190       3200
L1RP            GGAAAAGAGG AAGTCAAATT GTCCCTGTTT GCAGACGACA TGATTGTTTA
ORFeus-Hs       GGCAAGGAGG AGGTGAAGCT GAGCCTGTTC GCCGACGACA TGATCGTGTA
                ** ** **** * ** **  * *  ******  ** ******* **** ** **

                ....|....| ....|....| ....|....| ....|....| ....|....|
                      3210       3220       3230       3240       3250
L1RP            TCTAGAAAAC CCCATCGTCT CAGCCCAAAA TCTCCTTAAG CTGATAAGCA
ORFeus-Hs       CCTGGAGAAC CCCATCGTGA GCGCCCAGAA CCTGCTGAAG CTGATCAGCA
                 ** ** *** ********     ***** **  ** ** *** ***** ****

                ....|....| ....|....| ....|....| ....|....| ....|....|
                      3260       3270       3280       3290       3300
L1RP            ACTTCAGCAA AGTCTCAGGA TACAAAATCA ATGTACAAAA ATCACAAGCA
ORFeus-Hs       ACTTCAGCAA GGTGAGCGGC TACAAGATCA ACGTGCAGAA GAGCCAGGCC
                **********  **    **  ***** **** * ** ** **     ** **

                ....|....| ....|....| ....|....| ....|....| ....|....|
                      3310       3320       3330       3340       3350
L1RP            TTCTTATACA CCAACAACAG ACAAACAGAG AGCCAAATCA TGGGTGAACT
ORFeus-Hs       TTCCTGTACA CCAACAACCG CCAGACCGAG AGCCAGATCA TGGGCGAGCT
                *** * **** ******** *  ** ** *** ***** **** **** ** **

                ....|....| ....|....| ....|....| ....|....| ....|....|
                      3360       3370       3380       3390       3400
L1RP            CCCATTCACA ATTGCTTCAA AGAGAATAAA ATACCTAGGA ATCCAACTTA
ORFeus-Hs       GCCCTTCACC ATCGCTAGCA AGCGCATCAA GTACCTGGGC ATCCAGCTGA
                 ** *****  ** ***   * ** * ** **  ***** **  ***** ** *
                              Nhe I

                ....|....| ....|....| ....|....| ....|....| ....|....|
                      3410       3420       3430       3440       3450
L1RP            CAAGGGATGT GAAGGACCTC TTCAAGGAGA ACTACAAACC ACTGCTCAAG
ORFeus-Hs       CCCGCGACGT GAAGGACCTG TTCAAGGAGA ACTACAAGCC CCTGCTGAAG
                *  * ** ** *********  ********** ******* **  ***** ***

                ....|....| ....|....| ....|....| ....|....| ....|....|
                      3460       3470       3480       3490       3500
L1RP            GAAATAAAAG AGGAGACAAA CAAATGGAAG AACATTCCAT GCTCATGGGT
ORFeus-Hs       GAGATCAAGG AGGAGACCAA CAAGTGGAAG AACATCCCCT GCAGCTGGGT
                ** ** ** * ******* ** *** ****** ***** ** * **   *****

                ....|....| ....|....| ....|....| ....|....| ....|....|
                      3510       3520       3530       3540       3550
L1RP            AGGAAGAATC AATATCGTGA AAATGGCCAT ACTGCCCAAG GTAATTTACA
ORFeus-Hs       GGGCCGCATC AACATCGTGA AGATGGCCAT CCTGCCCAAG GTGATCTACC
                 **  * *** ** ******* * ********  ********* ** ** ***

                ....|....| ....|....| ....|....| ....|....| ....|....|
                      3560       3570       3580       3590       3600
L1RP            GATTCAATGC CATCCCCATC AAGCTACCAA TGACTTTCTT CACAGAATTG
ORFeus-Hs       GCTTCAACGC CATCCCCATC AAGCTGCCCA TGACCTTCTT CACCGAGCTG
                * ***** ** ********** ***** ** * **** ***** *** **  **
```

```
              ....|....| ....|....| ....|....| ....|....| ....|....|
                      3610       3620       3630       3640       3650
L1RP          GAAAAAACTA CTTTAAAGTT CATATGGAAC CAAAAAAGAG CCCGCATTGC
ORFeus-Hs     GAGAAGACCA CCCTGAAGTT CATCTGGAAC CAGAAGCGCG CCCGCATCGC
              ** ** ** * *  * ***** *** ****** ** **  * * ******* **

              ....|....| ....|....| ....|....| ....|....| ....|....|
                      3660       3670       3680       3690       3700
L1RP          CAAGTCAATC CTAAGCCAAA AGAACAAAGC TGGAGGCATC ACACTACCTG
ORFeus-Hs     CAAGAGCATC CTGAGCCAGA AGAACAAGGC CGGCGGCATC ACCCTGCCCG
              ****   *** ** ***** * ******* **  ** ****** ** ** ** *

              ....|....| ....|....| ....|....| ....|....| ....|....|
                      3710       3720       3730       3740       3750
L1RP          ACTTCAAACT ATACTACAAG GCTACAGTAA CCAAAACAGC ATGGTACTGG
ORFeus-Hs     ACTTCAAGCT GTACTACAAG GCCACCGTGA CCAAGACCGC CTGGTACTGG
              ******* **  ********* ** ** ** * **** ** **  *********

              ....|....| ....|....| ....|....| ....|....| ....|....|
                      3760       3770       3780       3790       3800
L1RP          TACCAAAACA GAGATATAGA TCAATGGAAC AGAACAGAGC CCTCAGAAAT
ORFeus-Hs     TACCAGAACC GCGATATCGA CCAGTGGAAC CGCACCGAGC CCAGCGAGAT
              ***** ***  * ***** **  ** ******  * ** **** **    ** **
                           EcoR V

              ....|....| ....|....| ....|....| ....|....| ....|....|
                      3810       3820       3830       3840       3850
L1RP          AATGCCGCAT ATCTACAACT ATCTGATCTT TGACAAACCT GAGAAAAACA
ORFeus-Hs     CATGCCCCAC ATCTACAACT ACCTGATCTT CGACAAGCCC GAGAAGAACA
               ***** **  ********** * ********  ***** **  ***** ****

              ....|....| ....|....| ....|....| ....|....| ....|....|
                      3860       3870       3880       3890       3900
L1RP          AGCAATGGGG AAAGGATTCC CTATTTAATA AATGGTGCTG GGAAAACTGG
ORFeus-Hs     AGCAGTGGGG CAAGGACAGC CTGTTCAACA AGTGGTGCTG GGAGAACTGG
              **** *****  *****    * ** ** ** * * ******** *** ******

              ....|....| ....|....| ....|....| ....|....| ....|....|
                      3910       3920       3930       3940       3950
L1RP          CTAGCCATAT GTAGAAAGCT GAAACTGGAT CCCTTCCTTA CACCTTATAC
ORFeus-Hs     CTGGCCATCT GCCGCAAGCT GAAGCTGGAT CCCTTCCTGA CCCCCTACAC
              ** ***** * *  * ***** *** ****** ******** * * ** ** **
                                        BamH I

              ....|....| ....|....| ....|....| ....|....| ....|....|
                      3960       3970       3980       3990       4000
L1RP          AAAAATCAAT TCAAGATGGA TTAAAGATTT AAACGTTAAA CCTAAAACCA
ORFeus-Hs     CAAGATCAAC AGCCGCTGGA TCAAGGACCT GAACGTGAAG CCCAAGACCA
               ** *****       * **** * ** **  *  ***** **  ** ** ****

              ....|....| ....|....| ....|....| ....|....| ....|....|
                      4010       4020       4030       4040       4050
L1RP          TAAAAACCCT AGAAGAAAAC CTAGGCATTA CCATTCAGGA CATAGGCGTG
ORFeus-Hs     TCAAGACCCT GGAGGAGAAC CTGGGCATCA CCATCCAGGA CATCGGCGTG
              * ** *****  ** ** *** ** ***** * **** ***** *** ******
```

```
            ....|....| ....|....| ....|....| ....|....| ....|....|
                   4060       4070       4080       4090       4100
L1RP        GGCAAGGACT TCATGTCCAA AACACCAAAA GCAATGGCAA CAAAAGACAA
ORFeus-Hs   GGCAAGGACT TCATGAGCAA GACCCCCAAG GCCATGGCCA CCAAGGACAA
            ********** *****  ***  ** ** **  ** ***** * * ** *****

            ....|....| ....|....| ....|....| ....|....| ....|....|
                   4110       4120       4130       4140       4150
L1RP        AATTGACAAA TGGGATCTAA TTAAACTAAA GAGCTTCTGC ACAGCAAAAG
ORFeus-Hs   GATCGACAAG TGGGACCTGA TCAAGCTGAA GAGCTTCTGC ACCGCCAAGG
             ** *****  ***** ** * * ** ** ** ********** ** ** ** *

            ....|....| ....|....| ....|....| ....|....| ....|....|
                   4160       4170       4180       4190       4200
L1RP        AAACTACCAT CAGAGTGAAC AGGCAACCTA CAACATGGGA GAAAATTTTT
ORFeus-Hs   AGACCACCAT CCGCGTGAAC CGCCAGCCCA CCACCTGGGA GAAGATCTTC
            * ** ***** * * ******  * ** ** * * ** ***** *** ** ** 

            ....|....| ....|....| ....|....| ....|....| ....|....|
                   4210       4220       4230       4240       4250
L1RP        GCAACCTACT CATCTGACAA AGGGCTAATA TCCAGAATCT ACAATGAACT
ORFeus-Hs   GCCACCTACA GCAGCGACAA GGGCCTGATC AGCCGCATCT ACAACGAGCT
            ** ******       *****  ** ** **    * * **** **** ** **

            ....|....| ....|....| ....|....| ....|....| ....|....|
                   4260       4270       4280       4290       4300
L1RP        CAAACAAATT TACAAGAAAA AAACAAACAA CCCCATCAAA AAGTGGGCGA
ORFeus-Hs   GAAGCAGATC TACAAGAAGA AGACCAACAA CCCCATCAAG AAGTGGGCCA
             ** ** **  ******** * * ** ***** *********  ******** *

            ....|....| ....|....| ....|....| ....|....| ....|....|
                   4310       4320       4330       4340       4350
L1RP        AGGACATGAA CAGACACTTC TCAAAAGAAG ACATTTATGC AGCCAAAAAA
ORFeus-Hs   AGGACATGAA CCGCCACTTC AGCAAGGAGG ACATCTACGC CGCCAAGAAG
            ********** * * ******    ** ** * **** ** **  ***** ** 

            ....|....| ....|....| ....|....| ....|....| ....|....|
                   4360       4370       4380       4390       4400
L1RP        CACATGAAGA AATGCTCATC ATCACTGGCC ATCAGAGAAA TGCAAATCAA
ORFeus-Hs   CATATGAAGA AGTGCAGCAG CAGCCTGGCC ATCCGCGAGA TGCAGATCAA
            ** ******* * ***          ****** *** * ** * **** *****

            ....|....| ....|....| ....|....| ....|....| ....|....|
                   4410       4420       4430       4440       4450
L1RP        AACCACTATG AGATATCATC TCACACCAGT TAGAATGGCA ATCATTAAAA
ORFeus-Hs   GACCACCATG CGCTACCACC TGACCCCCGT GCGCATGGCC ATCATCAAGA
             ***** ***  * ** ** * * ** ** **   * *****  ***** ** *

            ....|....| ....|....| ....|....| ....|....| ....|....|
                   4460       4470       4480       4490       4500
L1RP        AGTCAGGAAA CAACAGGTGC TGGAGAGGAT GCGGAGAAAT AGGAACACTT
ORFeus-Hs   AGAGCGGCAA CAACCGCTGC TGGCGCGGCT GCGGCGAGAT CGGCACCCTG
            **   ** ** **** * *** *** * ** * **** ** **  ** ** ** 
```

```
               ....|....| ....|....| ....|....| ....|....| ....|....|
                        4510       4520       4530       4540       4550
L1RP           TTACACTGTT GGTGGGACTG TAAACTAGTT CAACCATTGT GGAAGTCAGT
ORFeus-Hs      CTGCACTGCT GGTGGGACTG CAAGCTGGTG CAGCCCCTGT GGAAGAGCGT
                * ***** * **********  ** ** **  ** **  *** *****   **

               ....|....| ....|....| ....|....| ....|....| ....|....|
                        4560       4570       4580       4590       4600
L1RP           GTGGCGATTC CTCAGGGATC TAGAACTAGA AATACCATTT GACCCAGCCA
ORFeus-Hs      GTGGCGCTTC CTGCGCGACC TGGAGCTGGA GATCCCCTTC GACCCCGCCA
               ****** *** **  * ** * * ** ** **  ** ** **  ***** ****

               ....|....| ....|....| ....|....| ....|....| ....|....|
                        4610       4620       4630       4640       4650
L1RP           TCCCATTACT GGGTATATAC CCAAATGAGT ATAAATCATG CTGCTATAAA
ORFeus-Hs      TCCCCCTGCT GGGCATCTAC CCCAACGAGT ACAAGAGCTG CTGCTACAAG
               ****  * ** *** ** *** ** ** **** * **    ** ****** **

               ....|....| ....|....| ....|....| ....|....| ....|....|
                        4660       4670       4680       4690       4700
L1RP           GACACATGCA CACGTATGTT TATTGCGGCA CTATTCACAA TAGCAAAGAC
ORFeus-Hs      GACACCTGCA CCCGCATGTT CATCGCCGCC CTGTTCACCA TCGCCAAGAC
               ***** **** * ** *****  ** ** **  ** ***** * * ** *****

               ....|....| ....|....| ....|....| ....|....| ....|....|
                        4710       4720       4730       4740       4750
L1RP           TTGGAACCAA CCCAAATGTC CAACAATGAT AGACTGGATT AAGAAAATGT
ORFeus-Hs      CTGGAACCAG CCCAAGTGCC CCACCATGAT CGATTGGATC AAGAAGATGT
                ********  ***** ** * * ** *****  ** *****  ***** ****
                                                Cla I

               ....|....| ....|....| ....|....| ....|....| ....|....|
                        4760       4770       4780       4790       4800
L1RP           GGCACATATA CACCATGGAA TACTATGCAG CCATAAAAAA TGATGAGTTC
ORFeus-Hs      GGCACATCTA CACCATGGAG TACTACGCCG CCATCAAGAA CGACGAGTTC
               ******* ** *********  ***** ** * **** ** **  ** ******

               ....|....| ....|....| ....|....| ....|....| ....|....|
                        4810       4820       4830       4840       4850
L1RP           ATATCCTTTG TAGGGACATG GATGAAATTG GAAACCATCA TTCTCAGTAA
ORFeus-Hs      ATCAGCTTCG TGGGCACCTG GATGAAGCTG GAGACCATCA TCCTGAGCAA
               **   *** * * ** ** ** ******  ** ** ******* * ** ** **

               ....|....| ....|....| ....|....| ....|....| ....|....|
                        4860       4870       4880       4890       4900
L1RP           ACTATCGCAA GAACAAAAAA CCAAACACCG CATATTCTCA CTCATAGGTG
ORFeus-Hs      GCTGAGCCAG GAGCAGAAGA CCAAGCACCG CATCTTCAGC CTGATCGGCG
                **    ** ** ** ** * **** ***** *** ***    ** ** ** *

              ORF2 stop
               ....|...

L1RP           GGAATTGA
ORFeus-Hs      GCAACTGA
               * ** ***
```

Supplementary Figure 2

CMV start

```
                ....|....| ....|....| ....|....| ....|....| ....|....|
                        10         20         30         40         50
CMV             GTTGACATTG ATTATTGACT AGTTATTAAT AGTAATCAAT TACGGGGTCA
CMV+5' UTR      GTTGACATTG ATTATTGACT AGTTATTAAT AGTAATCAAT TACGGGGTCA
5’ UTR          ---------- ---------- ---------- ---------- ----------

                ....|....| ....|....| ....|....| ....|....| ....|....|
                        60         70         80         90        100
CMV             TTAGTTCATA GCCCATATAT GGAGTTCCGC GTTACATAAC TTACGGTAAA
CMV+5' UTR      TTAGTTCATA GCCCATATAT GGAGTTCCGC GTTACATAAC TTACGGTAAA
5’ UTR          ---------- ---------- ---------- ---------- ----------

                ....|....| ....|....| ....|....| ....|....| ....|....|
                       110        120        130        140        150
CMV             TGGCCCGCCT GGCTGACCGC CCAACGACCC CCGCCCATTG ACGTCAATAA
CMV+5' UTR      TGGCCCGCCT GGCTGACCGC CCAACGACCC CCGCCCATTG ACGTCAATAA
5’ UTR          ---------- ---------- ---------- ---------- ----------

                ....|....| ....|....| ....|....| ....|....| ....|....|
                       160        170        180        190        200
CMV             TGACGTATGT TCCCATAGTA ACGCCAATAG GGACTTTCCA TTGACGTCAA
CMV+5' UTR      TGACGTATGT TCCCATAGTA ACGCCAATAG GGACTTTCCA TTGACGTCAA
5’ UTR          ---------- ---------- ---------- ---------- ----------

                ....|....| ....|....| ....|....| ....|....| ....|....|
                       210        220        230        240        250
CMV             TGGGTGGAGT ATTTACGGTA AACTGCCCAC TTGGCAGTAC ATCAAGTGTA
CMV+5' UTR      TGGGTGGAGT ATTTACGGTA AACTGCCCAC TTGGCAGTAC ATCAAGTGTA
5’ UTR          ---------- ---------- ---------- ---------- ----------

                ....|....| ....|....| ....|....| ....|....| ....|....|
                       260        270        280        290        300
CMV             TCATATGCCA AGTCCGCCCC CTATTGACGT CAATGACGGT AAATGGCCCG
CMV+5' UTR      TCATATGCCA AGTCCGCCCC CTATTGACGT CAATGACGGT AAATGGCCCG
5’ UTR          ---------- ---------- ---------- ---------- ----------

                ....|....| ....|....| ....|....| ....|....| ....|....|
                       310        320        330        340        350
CMV             CCTGGCATTA TGCCCAGTAC ATGACCTTAC GGGACTTTCC TACTTGGCAG
CMV+5' UTR      CCTGGCATTA TGCCCAGTAC ATGACCTTAC GGGACTTTCC TACTTGGCAG
5’ UTR          ---------- ---------- ---------- ---------- ----------

                ....|....| ....|....| ....|....| ....|....| ....|....|
                       360        370        380        390        400
CMV             TACATCTACG TATTAGTCAT CGCTATTACC ATGGTGATGC GGTTTTGGCA
CMV+5' UTR      TACATCTACG TATTAGTCAT CGCTATTACC ATGGTGATGC GGTTTTGGCA
5’ UTR          ---------- ---------- ---------- ---------- ----------

                ....|....| ....|....| ....|....| ....|....| ....|....|
                       410        420        430        440        450
CMV             GTACACCAAT GGGCGTGGAT AGCGGTTTGA CTCACGGGGA TTTCCAAGTC
CMV+5' UTR      GTACACCAAT GGGCGTGGAT AGCGGTTTGA CTCACGGGGA TTTCCAAGTC
5’ UTR          ---------- ---------- ---------- ---------- ----------
```

```
                ....|....| ....|....| ....|....| ....|....| ....|....|
                        460        470        480        490        500
CMV             TCCACCCCAT TGACGTCAAT GGGAGTTTGT TTTGGCACCA AAATCAACGG
CMV+5' UTR      TCCACCCCAT TGACGTCAAT GGGAGTTTGT TTTGGCACCA AAATCAACGG
5′ UTR          ---------- ---------- ---------- ---------- ----------

                ....|....| ....|....| ....|....| ....|....| ....|....|
                        510        520        530        540        550
CMV             GACTTTCCAA AATGTCGTAA TAACCCCGCC CCGTTGACGC AAATGGGCGG
CMV+5' UTR      GACTTTCCAA AATGTCGTAA TAACCCCGCC CCGTTGACGC AAATGGGCGG
5′ UTR          ---------- ---------- ---------- ---------- ----------

                ....|....| ....|....| ....|....| ....|....| ....|....|
                        560        570        580        590        600
CMV             TAGGCGTGTA CGGTGGGAGG TCTATATAAG CAGAGCTCGT TTAGTGAACC
CMV+5' UTR      TAGGCGTGTA CGGTGGGAGG TCTATATAAG CAGAGCTCGT TTAGTGAACC
5′ UTR          ---------- ---------- ---------- ---------- ----------

                ....|....| ....|....| ....|....| ....|....| ....|....|
                        610        620        630        640        650
CMV             GTCAGATCTC TAGAAGCTGG GTACCAGCTG CTAGCAAGCT TGCTAGCGGC
CMV+5' UTR      GTCAGATCTC TAGAAGCTGG GTACCAGCTG CTAGCAAGCT TGCTAGCGGC
5′ UTR          ---------- ---------- ---------- ---------- ----------

                       Kozak sequence
                ....|....| ....|....| ....|....| ....|....| ....|....|
                        660        670        680        690        700
CMV             CGCGTTTAAA CTTAATTAAG CCACC ORF1 start..... ..........
CMV+5' UTR      CGCTCTAGCC CTGGAATGTG TATTAAGACT GTAAGGTGGG GGGGAGGAGC
5′ UTR          ---TCTAGCC CTGGAATGTG TATTAAGACT GTAAGGTGGG GGGGAGGAGC
                                                      |
                                                 5' UTR start

                ....|....| ....|....| ....|....| ....|....| ....|....|
                        710        720        730        740        750
CMV             .......... .......... .......... .......... ..........
CMV+5' UTR      CAAGATGGCC GAATAGGAAC AGCTCCGGTC TACAGCTCCC AGCGTGAGCG
5′ UTR          CAAGATGGCC GAATAGGAAC AGCTCCGGTC TACAGCTCCC AGCGTGAGCG

                ....|....| ....|....| ....|....| ....|....| ....|....|
                        760        770        780        790        800
CMV             .......... .......... .......... .......... ..........
CMV+5' UTR      ACGCAGAAGA CGGTGATTTC TGCATTTCCA TCTGAGGTAC CGGGTTCATC
5′ UTR          ACGCAGAAGA CGGTGATTTC TGCATTTCCA TCTGAGGTAC CGGGTTCATC

                ....|....| ....|....| ....|....| ....|....| ....|....|
                        810        820        830        840        850
CMV             .......... .......... .......... .......... ..........
CMV+5' UTR      TCACTAGGGA GTGCCAGACA GTGGGCGCAG GCCAGTGTGT GTGCGCACCG
5′ UTR          TCACTAGGGA GTGCCAGACA GTGGGCGCAG GCCAGTGTGT GTGCGCACCG

                ....|....| ....|....| ....|....| ....|....| ....|....|
                        860        870        880        890        900
CMV             .......... .......... .......... .......... ..........
CMV+5' UTR      TGCGCGAGCC GAAGCAGGGC GAGGCATTGC CTCACCTGGG AAGCGCAAGG
5′ UTR          TGCGCGAGCC GAAGCAGGGC GAGGCATTGC CTCACCTGGG AAGCGCAAGG
```

```
               ....|....| ....|....| ....|....| ....|....| ....|....|
                       910        920        930        940        950
CMV            .......... .......... .......... .......... ..........
CMV+5' UTR     GGTCAGGGAG TTCCCTTTCC GAGTCAAAGA AAGGGGTGAC GGACGCACCT
5’ UTR         GGTCAGGGAG TTCCCTTTCC GAGTCAAAGA AAGGGGTGAC GGACGCACCT

               ....|....| ....|....| ....|....| ....|....| ....|....|
                       960        970        980        990       1000
CMV            .......... .......... .......... .......... ..........
CMV+5' UTR     GGAAAATCGG GTCACTCCCA CCCGAATATT GCGCTTTTCA GACCGGCTTA
5’ UTR         GGAAAATCGG GTCACTCCCA CCCGAATATT GCGCTTTTCA GACCGGCTTA

               ....|....| ....|....| ....|....| ....|....| ....|....|
                      1010       1020       1030       1040       1050
CMV            .......... .......... .......... .......... ..........
CMV+5` UTR     AGAAACGGCG CACCACGAGA CTATATCCCG CACCTGGCTC GGAGGGTCCT
5’ UTR         AGAAACGGCG CACCACGAGA CTATATCCCG CACCTGGCTC GGAGGGTCCT

               ....|....| ....|....| ....|....| ....|....| ....|....|
                      1060       1070       1080       1090       1100
CMV            .......... .......... .......... .......... ..........
CMV+5' UTR     ACGCCCACGG AATCTCGCTG ATTGCTAGCA CAGCAGTCTG AGATCAAACT
5’ UTR         ACGCCCACGG AATCTCGCTG ATTGCTAGCA CAGCAGTCTG AGATCAAACT

               ....|....| ....|....| ....|....| ....|....| ....|....|
                      1110       1120       1130       1140       1150
CMV            .......... .......... .......... .......... ..........
CMV+5' UTR     GCAAGGCGGC AACGAGGCTG GGGGAGGGGC GCCCGCCATT GCCCAGGCTT
5’ UTR         GCAAGGCGGC AACGAGGCTG GGGGAGGGGC GCCCGCCATT GCCCAGGCTT

               ....|....| ....|....| ....|....| ....|....| ....|....|
                      1160       1170       1180       1190       1200
CMV            .......... .......... .......... .......... ..........
CMV+5' UTR     GCTTAGGTAA ACAAAGCAGC AGGGAAGCTC GAACTGGGTG GAGCCCACCA
5’ UTR         GCTTAGGTAA ACAAAGCAGC AGGGAAGCTC GAACTGGGTG GAGCCCACCA

               ....|....| ....|....| ....|....| ....|....| ....|....|
                      1210       1220       1230       1240       1250
CMV            .......... .......... .......... .......... ..........
CMV+5` UTR     CAGCTCAAGG AGGCCTGCCT GCCTCTGTAG GCTCCACCTC TGGGGGCAGG
5’ UTR         CAGCTCAAGG AGGCCTGCCT GCCTCTGTAG GCTCCACCTC TGGGGGCAGG

               ....|....| ....|....| ....|....| ....|....| ....|....|
                      1260       1270       1280       1290       1300
CMV            .......... .......... .......... .......... ..........
CMV+5' UTR     GCACAGACAA ACAAAAAGAC AGCAGTAACC TCTGCAGACT TAAGTGTCCC
5’ UTR         GCACAGACAA ACAAAAAGAC AGCAGTAACC TCTGCAGACT TAAGTGTCCC

               ....|....| ....|....| ....|....| ....|....| ....|....|
                      1310       1320       1330       1340       1350
CMV            .......... .......... .......... .......... ..........
CMV+5' UTR     TGTCTGACAG CTTTGAAGAG AGCAGTGGTT CTCCCAGCAC GCAGCTGGAG
5’ UTR         TGTCTGACAG CTTTGAAGAG AGCAGTGGTT CTCCCAGCAC GCAGCTGGAG
```

```
                ....|....| ....|....| ....|....| ....|....| ....|....|
                        1360      1370      1380      1390      1400
CMV             .......... .......... .......... .......... ..........
CMV+5' UTR      ATCTGAGAAC GGGCAGACTG CCTCCTCAAG TGGGTCCCTG ACCCCTGACC
5’ UTR          ATCTGAGAAC GGGCAGACTG CCTCCTCAAG TGGGTCCCTG ACCCCTGACC

                ....|....| ....|....| ....|....| ....|....| ....|....|
                        1410      1420      1430      1440      1450
CMV             .......... .......... .......... .......... ..........
CMV+5' UTR      CCCGAGCAGC CTAACTGGGA GGCACCCCCC AGCAGGGGCA CACTGACACC
5’ UTR          CCCGAGCAGC CTAACTGGGA GGCACCCCCC AGCAGGGGCA CACTGACACC

                ....|....| ....|....| ....|....| ....|....| ....|....|
                        1460      1470      1480      1490      1500
CMV             .......... .......... .......... .......... ..........
CMV+5' UTR      TCACACGGCA GGGTATTCCA ACAGACCTGC AGCTGAGGGT CCTGTCTGTT
5’ UTR          TCACACGGCA GGGTATTCCA ACAGACCTGC AGCTGAGGGT CCTGTCTGTT

                ....|....| ....|....| ....|....| ....|....| ....|....|
                        1510      1520      1530      1540      1550
CMV             .......... .......... .......... .......... ..........
CMV+5' UTR      AGAAGGAAAA CTAACAACCA GAAAGGACAT CTACACCGAA AACCCATCTG
5’ UTR          AGAAGGAAAA CTAACAACCA GAAAGGACAT CTACACCGAA AACCCATCTG

                ....|....| ....|....| ....|....| ....|....| ....|....
                        1560      1570      1580      1590
CMV             .......... .......... .......... .......... .........
CMV+5' UTR      TACATCACCA TCATCAAAGA CCAAAAGTAG ATAAAACCAC AAAG ORF1 start
5’ UTR          TACATCACCA TCATCAAAGA CCAAAAGTAG ATAAAACCAC AAAG ORF1 start
```

# Supplementary Figure 3

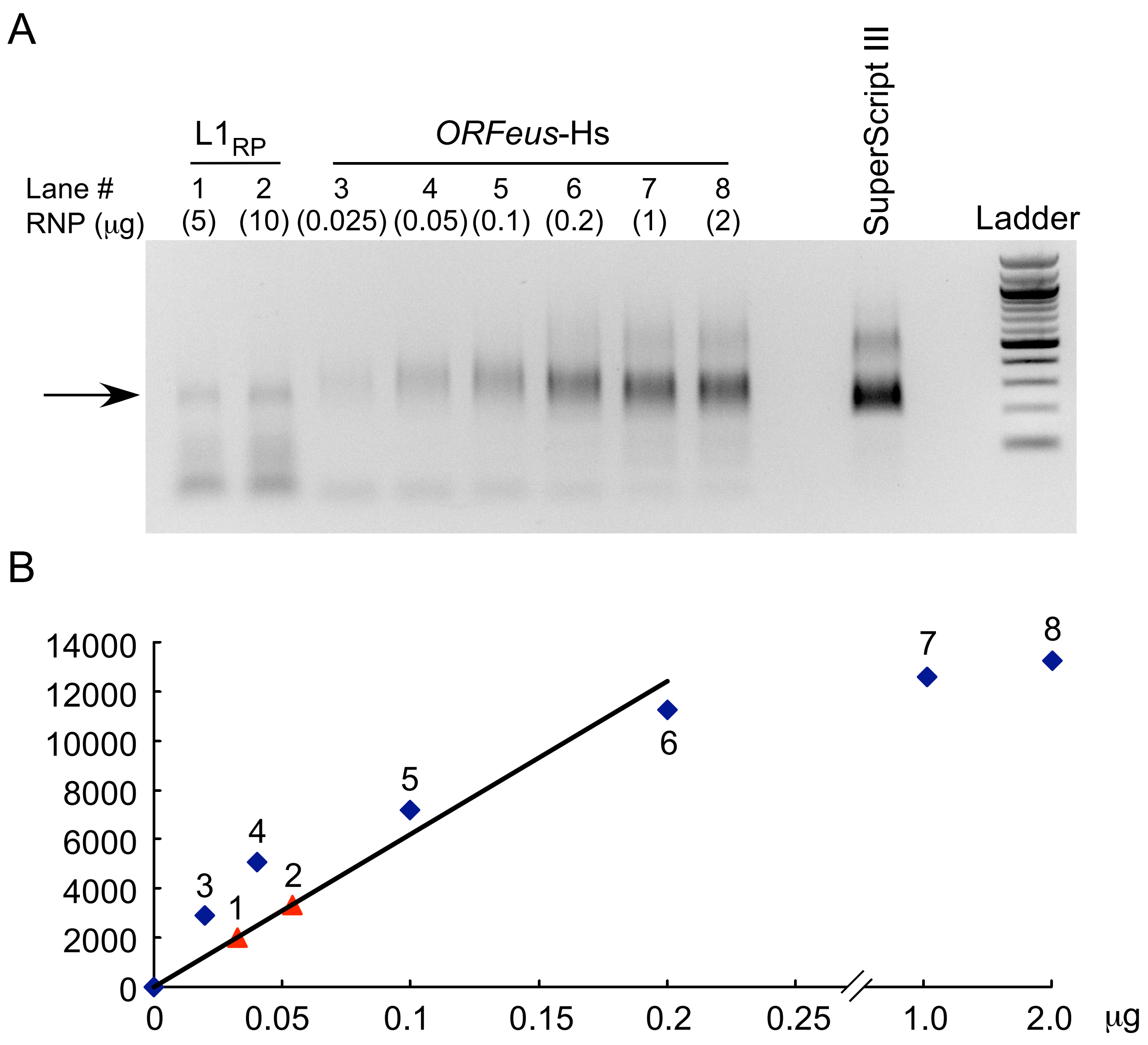

# Supplementary Figure 4

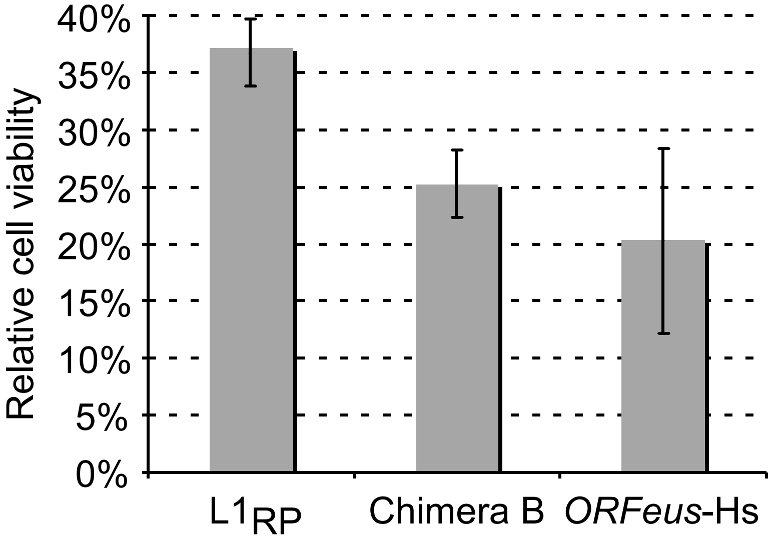

Supplement: Additional file 1 — Supplementary Figures 1-4. (1) Alignment of native human L1RP with ORFeus-Hs. BioEdit was used to create a nucleic acid alignment of native human L1 and ORFeus-Hs, starting at the ATG of open reading frame (ORF)1 and ending at the stop codon of ORF2. For these sequences, the base composition of L1RP is 40% A (1998), 21% C (1047), 19% G (906), 20% T (967). The base composition of ORFeus-HS is 27% A (1322), 34% C (1648), 27% (1314) G, 12% T (624). L1RP (Genbank accession number AF148856) was used as the sequence for native human L1. Identities are marked with asterisks. Start and stop codons of ORF1 and ORF2, restriction sites used to clone building blocks (MfeI, BsmBI, AscI, AgeI, BstBI, NruI, XmaI, MluI, NheI, EcoRV, NdeI, ClaI, XhoI) and make native/synthetic chimeras (PmlI, EcoRI and BamHI) are highlighted in gray boxes. (2) Alignment of different promoters used in this study. Kozak sequence and boundaries of cytomegalovirus (CMV) and the 5' untranslated region (UTR) are highlighted. (3) Quantification of L1 element amplification protocol (LEAP). (A) LEAP was performed using a ribonucleoprotein (RNP) preparation with the indicated amount, and an equal amount of PCR product was loaded onto a 1.5% agarose gel. The arrow indicates the mobility of LEAP PCR product on the gel. (B) The density of the bands was quantified using the Multi-Gauge program and plotted as a function of the amount of ORFeus-Hs RNP. A trend line was drawn using values from lane 3 to lane 6 and the X-axis values from lane 1 to lane 2 (0.03 and 0.05 μg respectively) were calculated based on the trend line. Blue diamond = data from ORFeus-Hs RNP (lanes 3 to 8); red triangle = data from L1RP RNP (lanes 1 and 2). X-axis, amount of ORFeus-Hs RNP in uG; Y-axis, pixel value. (4) Cell-viability assay. Relative cell viability is presented as the ratio of viable puromycin-resistant cells divided by total viable cells (without puromycin selection). Four independent transfections were performed for each cons [file 1759-8753-2-2-S1.PDF]
