# Supplementary material for: Characterization of a synthetic human LINE-1 retrotransposon ORFeus-Hs
Source: Mob DNA. 2011 Feb 14;2:2. doi: 10.1186/1759-8753-2-2 (PMC3045867; doi:10.1186/1759-8753-2-2)
Supplement: Additional file 2 — Supplementary Tables. Tables 1 and 2. [file 1759-8753-2-2-S2.DOC]

Supplementary Table 1. L1 constructs used in this study.

| Construct | Backbone | CMV | 5’UTR | Kozak | ORF1 | ORF2 | 3’UTR | CTE | Marker |
| --- | --- | --- | --- | --- | --- | --- | --- | --- | --- |
| pLD223 | pCEP puro | Yes | No | Yes | Native | Native | Yes | Yes | *GFP-AI* |
| pWA172 | pCEP puro | Yes | No | Yes | Synthetic | Native-P-Synthetic | Yes | Yes | *GFP-AI* |
| pWA170 | pCEP puro | Yes | No | Yes | Synthetic | Native-E-Synthetic | Yes | Yes | *GFP-AI* |
| pWA176 | pCEP puro | Yes | No | Yes | Synthetic | Native-B-Synthetic | Yes | Yes | *GFP-AI* |
| pWA163 | pCEP puro | Yes | No | Yes | Synthetic | Synthetic | Yes | Yes | *GFP-AI* |
| pWA174 | pCEP puro | Yes | Yes | No | Native | Native | Yes | Yes | *GFP-AI* |
| pLD224 | pCEP puro | Yes | Yes | No | Synthetic | Native-P-Synthetic | Yes | Yes | *GFP-AI* |
| pLD227 | pCEP puro | Yes | Yes | No | Synthetic | Native-E-Synthetic | Yes | Yes | *GFP-AI* |
| pLD225 | pCEP puro | Yes | Yes | No | Synthetic | Native-B-Synthetic | Yes | Yes | *GFP-AI* |
| pWA165 | pCEP puro | Yes | Yes | No | Synthetic | Synthetic | Yes | Yes | *GFP-AI* |
| pLD143 | pCEP puro | No | Yes | No | Native | Native | Yes | Yes | *GFP-AI* |
| pLD252 | pCEP puro | No | Yes | No | Synthetic | Native-P-Synthetic | Yes | Yes | *GFP-AI* |
| pLD253 | pCEP puro | No | Yes | No | Synthetic | Native-E-Synthetic | Yes | Yes | *GFP-AI* |
| pLD254 | pCEP puro | No | Yes | No | Synthetic | Native-B-Synthetic | Yes | Yes | *GFP-AI* |
| pLD255 | pCEP puro | No | Yes | No | Synthetic | Synthetic | Yes | Yes | *GFP-AI* |
| pWA160 | pCEP puro | No | No | No | Synthetic | Synthetic | No | No | *GFP-AI* |
| pWA192 | pCEP puro | Yes | Yes | No | Native | Native | Yes | Yes | *Neo-AI* |
| pWA189 | pCEP puro | Yes | Yes | No | Synthetic | Native-P-Synthetic | Yes | Yes | *Neo-AI* |
| pWA188 | pCEP puro | Yes | Yes | No | Synthetic | Native-E-Synthetic | Yes | Yes | *Neo-AI* |
| pWA191 | pCEP puro | Yes | Yes | No | Synthetic | Native-B-Synthetic | Yes | Yes | *Neo-AI* |
| pWA193 | pCEP puro | Yes | Yes | No | Synthetic | Synthetic | Yes | Yes | *Neo-AI* |
| pWA200 | pCEP puro | Yes | No | Yes | Synthetic | Synthetic | No | No | *Neo-AI* |
| pWA182 | pCEP puro | Yes | No | Yes | Synthetic | Synthetic | Yes | No | *Neo-AI* |
| pWA183 | pCEP puro | Yes | No | Yes | Synthetic | Synthetic | Yes | Yes | *Neo-AI* |
| pWA180 | pCEP puro | Yes | Yes | No | Synthetic | Synthetic | No | No | *Neo-AI* |
| pWA184 | pCEP puro | Yes | Yes | No | Synthetic | Synthetic | Yes | No | *Neo-AI* |
| pWA199 | pCEP puro | Yes | Yes | No | Synthetic | Synthetic | Yes | Yes | *Neo-AI* |
| pWA181 | pCEP puro | Yes | Yes | Yes | Synthetic | Synthetic | No | No | *Neo-AI* |
| pWA185 | pCEP puro | Yes | Yes | Yes | Synthetic | Synthetic | Yes | No | *Neo-AI* |
| pWA186 | pCEP puro | Yes | Yes | Yes | Synthetic | Synthetic | Yes | Yes | *Neo-AI* |

Supplementary Table 2. Retrotransposition frequency of *ORFeus*-Hs constructs with *Neo-AI* marker.

|  | CMV+Kozak^a^ |
| --- | --- |
| Fully native | 1.0 ± 0.0 (pWA192) |
| Chimera-P | 0.7 ± 0.3 (pWA189) |
| Chimera-E | 1.8 ± 0.4 (pWA188) |
| Chimera-B | 5.4 ± 1.2 (pWA191) |
| *ORFeus*-Hs | 1.9 ± 0.2 (pWA193) |
| *ORFeus*-Hs (synthetic interORF) | 2.0 ± 0.1 (pWA206) |

^a^ fold changes of retrotransposition frequency relative to native L1_RP_ (pWA192). Data are mean of six independent experiments. ± standard error
